# Genomic resolution of linkages in carbon, nitrogen, and sulfur cycling among widespread estuary sediment bacteria

**DOI:** 10.1186/s40168-015-0077-6

**Published:** 2015-04-13

**Authors:** Brett J Baker, Cassandre Sara Lazar, Andreas P Teske, Gregory J Dick

**Affiliations:** Department of Marine Science, University of Texas-Austin, Marine Science Institute, 750 Channel View Dr., Port Aransas, TX 78373 USA; Department of Earth and Environmental Sciences, University of Michigan, 1100 N. University Ave., Ann Arbor, MI 48109 USA; Department of Marine Sciences, University of North Carolina, Chapel Hill, NC USA; Organic Geochemistry Group, MARUM Center for Marine Environmental Sciences, Department of Geosciences, University of Bremen, Bremen, Germany; Center for Computational Medicine and Bioinformatics, University of Michigan, Ann Arbor, MI USA

**Keywords:** Estuary, Sediment, Metagenome, Sulfur, Nitrogen, Carbon, Candidate phyla, Anaerobic respiration, Sulfate reduction

## Abstract

**Background:**

Estuaries are among the most productive habitats on the planet. Bacteria in estuary sediments control the turnover of organic carbon and the cycling of nitrogen and sulfur. These communities are complex and primarily made up of uncultured lineages, thus little is known about how ecological and metabolic processes are partitioned in sediments.

**Results:**

*De novo* assembly and binning resulted in the reconstruction of 82 bacterial genomes from different redox regimes of estuary sediments. These genomes belong to 23 bacterial groups, including uncultured candidate phyla (for example, KSB1, TA06, and KD3-62) and three newly described phyla (White Oak River (WOR)-1, WOR-2, and WOR-3). The uncultured phyla are generally most abundant in the sulfate-methane transition (SMTZ) and methane-rich zones, and genomic data predict that they mediate essential biogeochemical processes of the estuarine environment, including organic carbon degradation and fermentation. Among the most abundant organisms in the sulfate-rich layer are novel *Gammaproteobacteria* that have genes for the oxidation of sulfur and the reduction of nitrate and nitrite. Interestingly, the terminal steps of denitrification (NO_3_ to N_2_O and then N_2_O to N_2_) are present in distinct bacterial populations.

**Conclusions:**

This dataset extends our knowledge of the metabolic potential of several uncultured phyla. Within the sediments, there is redundancy in the genomic potential in different lineages, often distinct phyla, for essential biogeochemical processes. We were able to chart the flow of carbon and nutrients through the multiple geochemical layers of bacterial processing and reveal potential ecological interactions within the communities.

**Electronic supplementary material:**

The online version of this article (doi:10.1186/s40168-015-0077-6) contains supplementary material, which is available to authorized users.

## Background

Estuaries are dynamic environments where nutrient-rich river water mixes with shallow coastal and nutrient-rich deep ocean water. They are among the most productive environments on the planet [1]. Processes within estuaries also mediate the transfer of carbon from land to sea, release a considerable amount of CO_2_ to the atmosphere [2], and sequester carbon in sediments [3]. As this organic carbon is degraded, and oxygen is consumed, a variety of favorable anaerobic respiratory processes are mediated by sediment microorganisms that contribute to sulfur, nitrogen, and iron transformations. Thus, shallow estuary sediments and their microbial communities are a global hotspot for biogeochemical cycling.

Our ability to partition these processes and the underlying metabolic pathways among specific microbial groups have remained limited by the complexity and abundance of uncultured groups present in sediment communities. Metagenomic studies have characterized the genetic potential of marine sediment microbial communities (for example, see Biddle *et al.* [4]). However, because sequencing reads are often not assigned to specific taxa, such approaches typically do not link individual community members to metabolic pathways [5]. Further, culture-independent genomic reconstructions of estuary sediment microbial communities are lacking; thus little is known about uncultured communities in this complex microbial ecosystem.

To better understand the metabolic capabilities of uncultured bacteria, we obtained 262 Gbp of genomic sequence from sediment profiles from the White Oak River (WOR) estuary, North Carolina. Our sequencing effort focused on three key redox layers; the sulfate-rich, sulfate-methane transition (SMTZ), and methane-rich zones. This genomic dataset was assembled and binned to obtain partial and near-complete genomes and to reconstruct metabolic pathways of numerous community members. Reconstructing the metabolic capabilities of numerous bacterial groups in the sediment communities enabled us to identify their roles in carbon, iron, nitrogen, and sulfur cycling. This dataset provides a genomic road map of how biogeochemical processes are hypothesized to be partitioned in aquatic sediment bacterial communities.

## Results and discussion

### Genomic reconstruction and identification

**Figure 1:**
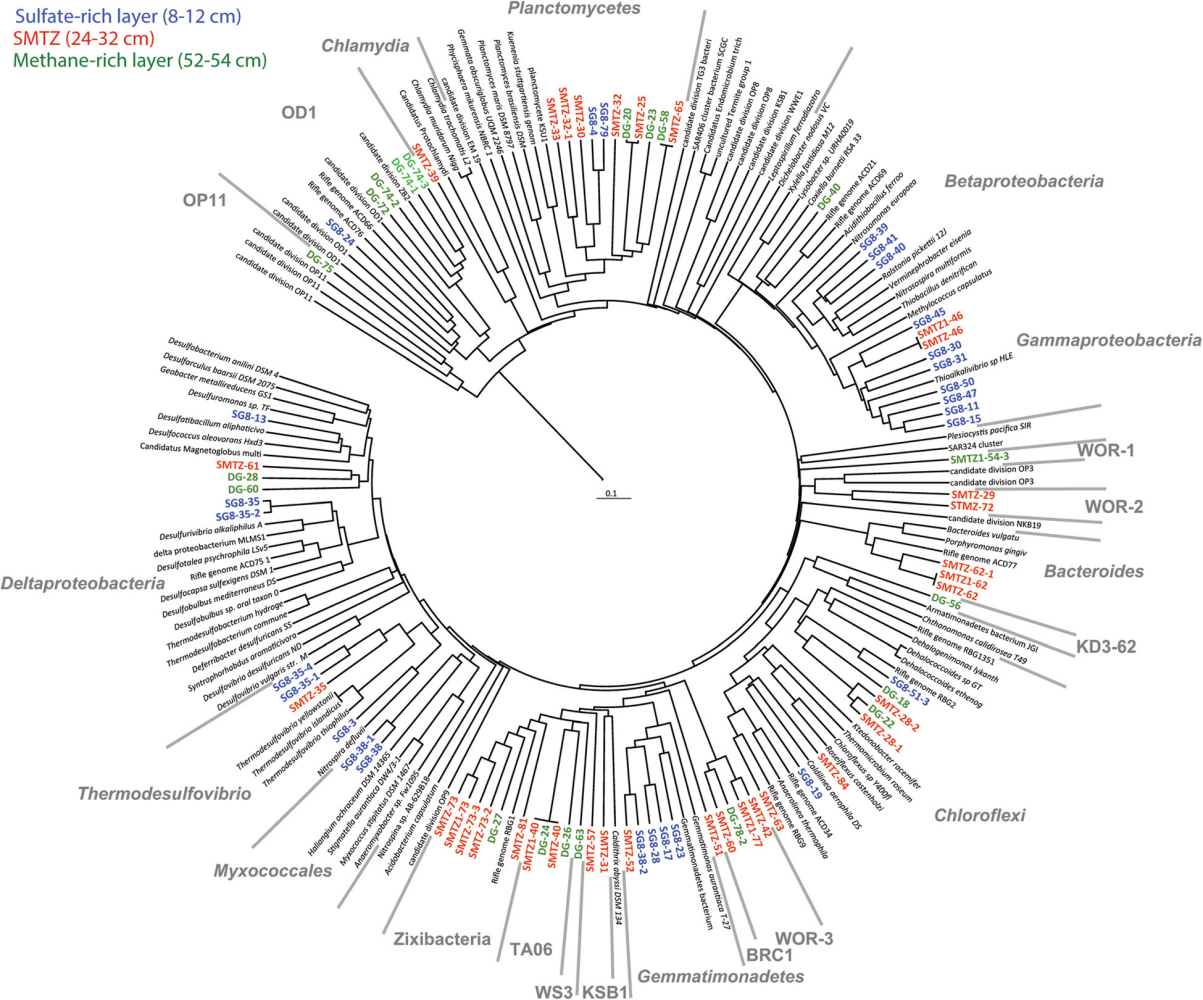
**Diversity of organisms from which genomic bins were reconstructed from the White Oak River sediments.** Phylogenetic tree inferred from 16 syntenous ribosomal protein genes present within genomic bins from the sediment metagenomic assemblies. Each sequence in bold is from one genomic bin. Genomic bins belonging to novel phyla for which no reference genomes are available (WOR-1, WOR-2, WOR-3, TA06, and KD3-62) were designated based on corresponding 16S rRNA gene phylogenetic analyses (Figure 2). The sample depths from which each of the bins were obtained are delineated by blue (shallow), green (SMTZ), and red (deep). The phylogeny was generated using the PhyML (maximum likelihood) method.

**Figure 2:**
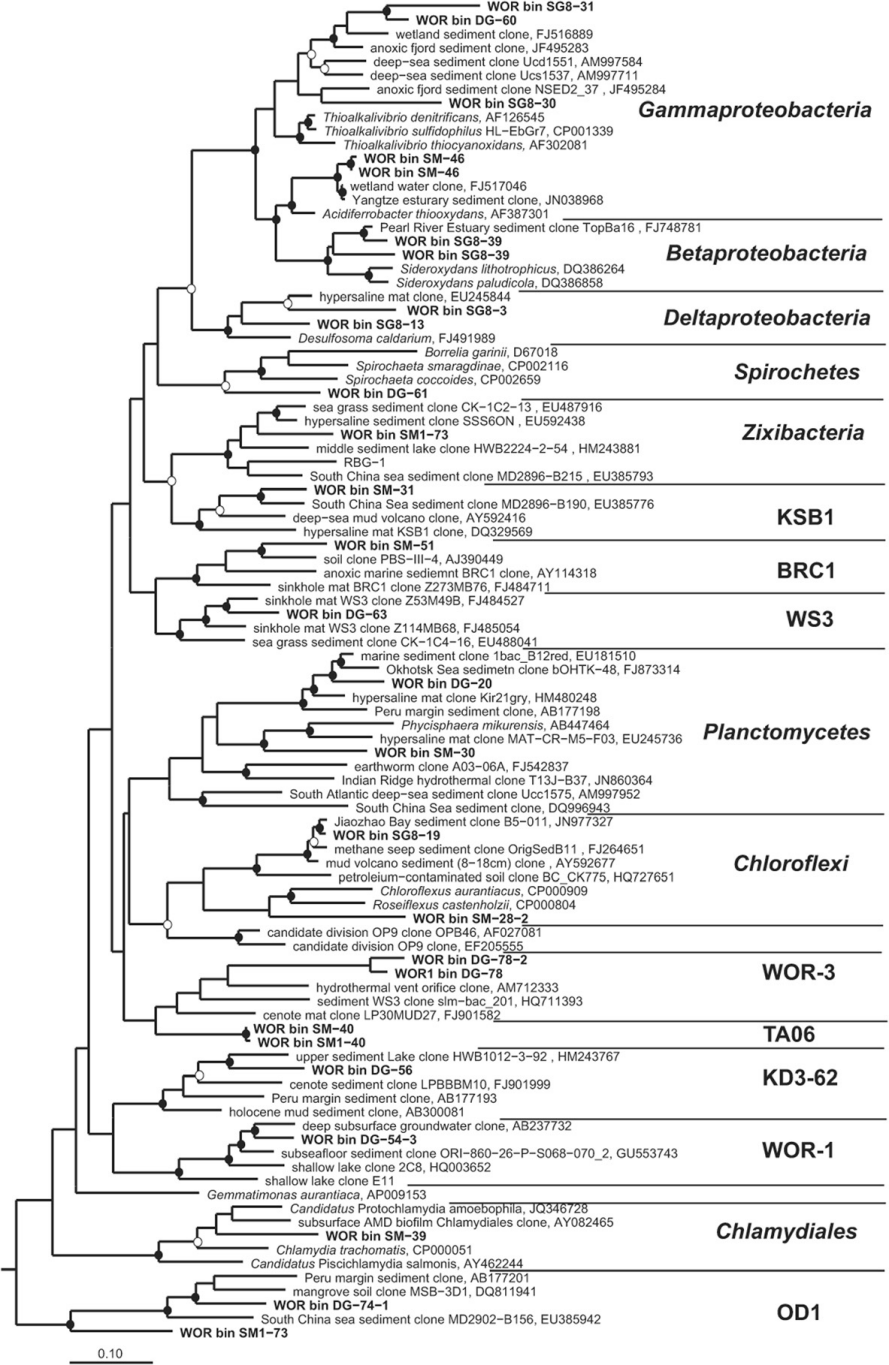
**Phylogenetic tree of 16S rRNA genes present in bacterial genomic bins.** Top hits from NCBI were included. Many of the White Oak River bacteria are most closely related to sequences recovered from other estuaries and coastal sediments. This tree was generated using the maximum likelihood method in the ARB alignment and phylogeny software package [59]. Closed circles represent maximum likelihood (RAxML, ARB package) bootstraps >75% and open circles are >50% values.

Sediment samples were collected with push cores at three adjacent mid-estuary locations. Since the distinct redox layers shared similar bacterial communities across the three sites, we combined genomic reads from sites 2 and 3 from the sulfate-rich zone (8 to 12 cm) and SMTZ (24 to 32 cm). The co-assembly of separate samples resulted in better assembly and greater coverage of the genomic bins. Due to the large number of SMTZ sequence reads, a separate assembly was generated from site 1 (26 to 30 cm). The methane-rich zone (52 to 54 cm) assembly was generated only from site 1. Statistics (total size, N50, number of ORFs, *etc*.) about these assemblies used for binning can be found in Additional file 1: Table S1. Subsequent binning by tetra-nucleotide frequency coupled with genomic coverage resulted in over 120 genomic bins of Bacteria (Additional file 1: Figure S1). Based on completeness, taxon coverage, and genomic novelty, 82 bins were chosen for detailed characterization of genome-encoded metabolic pathways. Of the bins, 26, 35, and 21 are from the sulfate-rich, SMTZ, and methane-rich zones, respectively. Of these genomes, 58 are estimated to be >70% complete and 32 are >80% complete, with minimal estimated contamination (average approximately 10% among them all) (Additional file 1: Table S2). Eight of the bins were found to contain more than one genome, by the presence of multiple single-copy genes [6]. Only 30 of these genomes contain 16S rRNA genes (>300 bp) likely due to fragmentation commonly seen among highly conserved genes in short-read assemblies [7]. Therefore, phylogenetic analysis of concatenated ribosomal proteins was also used to determine the taxonomic identities of the remaining bins (Figure 1) [8]. Ribosomal proteins belonging to novel phyla for which reference sequences were not available were identified by the presence of at least one 16S rRNA gene in the clade (Figure 2). The distribution of ribosomal proteins and other single-copy genes is detailed in Additional file 1: Table S3.

Several of the genomic bins belong to groups that are commonly identified in rRNA gene surveys of marine and estuarine sediments, including *Betaproteobacteria*, *Gammaproteobacteria*, *Deltaproteobacteria*, *Chloroflexi*, *Planctomycetes*, *Bacteroidetes*, *Gemmatimonadetes*, *Nitrospira*, *Chlamydiae*, and *Spirochetes* [9,10]. Many of the 16S rRNA genes are most similar to sequences from other estuaries (for example, Pearl River [11] and Yangtze estuary sediments) and marine sediments (for example, South China Sea [12]) (Figure 2). Genomes recovered from candidate phyla that are commonly identified in a variety of anoxic environments and marine sediments comprise OD1 (Parcubacteria), WS3 (Latescibacteria), TA06, Zixibacteria, and BRC1 (Additional file 1: Table S4). Two of the genomic bins belong to previously unnamed phylum-level lineages of 16S rRNA genes, mostly recovered from marine sediments, referred to as ‘WOR-1 and WOR-3’, for White Oak River groups. These bins (SMTZ1-54-3, DG-78, and DG-78-2) are deeply divergent from all currently recognized phyla based on 16S rRNA gene phylogeny (Figure 2) and concatenated ribosomal proteins (bins SMTZ-42, -60, SMTZ1-77, -54-3, and DG78-2, Figure 1). Based on ribosomal proteins, an additional phylogenetically distinct group (bins SMTZ-29 and SMTZ-72), which we will refer to here at ‘WOR-2’ was identified.

### Genomic abundance of community members in the sediment profile

To quantify the genomic abundance of community members in each zone, all reads were mapped to all genes for ribosomal protein S3 in the genome assemblies (Additional file 1: Figure S2). The assembly from shallow, sulfate-rich sediments was dominated by *Beta-*, *Gamma*-, and *Deltaproteobacteria* (including *Myxococcales*), *Bacteroidetes*, and *Nitrospira*. The SMTZ and the deeper methane-rich sediments were dominated by Archaea and *Chloroflexi* (Additional file 1: Figure S2), consistent with previous qPCR and rRNA slot blot results showing that bacterial dominance in surficial White Oak River estuarine sediments is considerably reduced and even reversed downcore [13]. The candidate bacterial phyla WS3, OD1, TA06, Zixibacteria, WOR-1, WOR-2, and WOR-3 were sufficiently abundant for genome reconstruction only in the deeper sediment layers.

### Organic carbon degradation and fermentation

**Figure 3:**
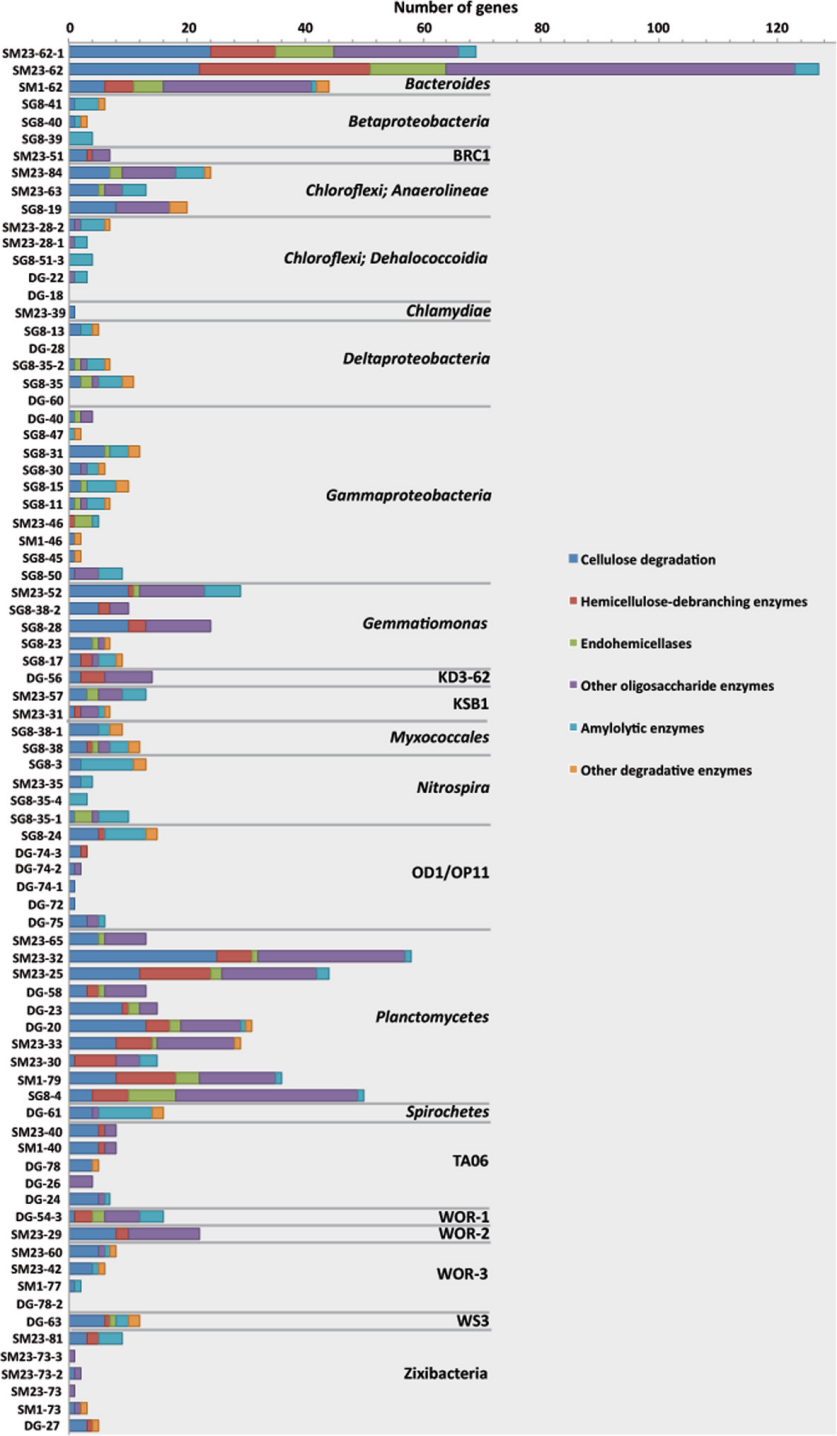
**Glycoside hydrolases (GH) identified by CAZy searches of the genomic bins.** GH families that contain enzymes that are not specifically involved in degradation were specifically identified by pfam or EC numbers in the annotations, based on Wrighton *et al.* [22].

Sedimentary microbial communities process the input of photosynthetic organic matter from the overlying water column and thus play a key role in the degradation of complex carbon substrates [14]. All the bacterial genomes present here were searched for carbohydrate-metabolizing enzymes using the ‘CAZy’ database [15]. Many of the genes in CAZy are involved in cellular maintenance processes; therefore, those that are specifically involved in hydrolysis of organic carbon based on previous studies [16] were identified. Generally, the genomes belonging to the *Chloroflexi* (specifically *Anaerolineae*), *Bacteroidetes*, *Gemmatimonadetes*, *Planctomycetes*, WOR-1, and WOR-2 phyla contained the broadest array of carbohydrate hydrolytic genes (Figure 3). Candidate phyla KSB1 and KD3-62 also have relatively high numbers of these genes. These organisms have a variety of cellulose, hemicellulose, and polysaccharide degradation genes suggesting a role in the initial degradation and hydrolysis of complex organic carbon compounds. Multiple genes are involved in the degradation of chitin, the long-chain polymer of N-acetylglucosamine, a major structural component of fungal and algal cell wells, and of arthropod exoskeletons. Endo-acting chitinase genes were identified in two of the WOR-3 bins (SMTZ-60 and SMTZ-42), KSB1 (or Cloacimonetes) bin SMTZ-31, and a variety of *Chloroflexi* and *Planctomyetes. N*-acetyl-glucosaminidase genes were found in several *Bacteroides*, *Gammaproteobacteria*, *Deltaproteobacteria*, and Zixibacteria genomes.

Proteins account for a large proportion of bioavailable carbon and nitrogen for sediment communities [17]. In multiple phyla, these resources are accessed via extracellular peptidases (Additional file 2: Table S5). The greatest numbers of peptidases were identified in the candidate phyla KSB1, WOR-1, WOR-2, and WOR-3, and in the *Bacteroidetes* and *Gemmatimonadetes*, suggesting that these community members are substantially involved in protein degradation along with sedimentary benthic Archaea [18].

Several of the *Chloroflexi* genomes (SG8-19, DG-18, SMTZ-63, and SMTZ-84) contain the *β*-oxidation pathway to generate acetyl-CoA from fatty acids and organic acids [8,19]. This capability is also present in genomes from both of the *Myxococcales* (SG8-38 and SG8-38-1), *Gemmatimonadetes* (SG8-23 and SG8-28), several *Gammaproteobacteria* (SG8-11, SG8-30, SG8-31, SG8-50, SM1-46, and SM23-46) and *Deltaproteobacteria* (SG8-13 and SM23-61), and the shallow sediment-dwelling *Betaproteobacteria*. Among the candidate phyla that contain the complete *β*-oxidation pathway are the BRC-1, and one of the KSB1 (SMTZ-57) genomic bins. A majority of genomes that have the *β*-oxidation pathway (60%) were primarily found in the shallow samples.

**Figure 4:**
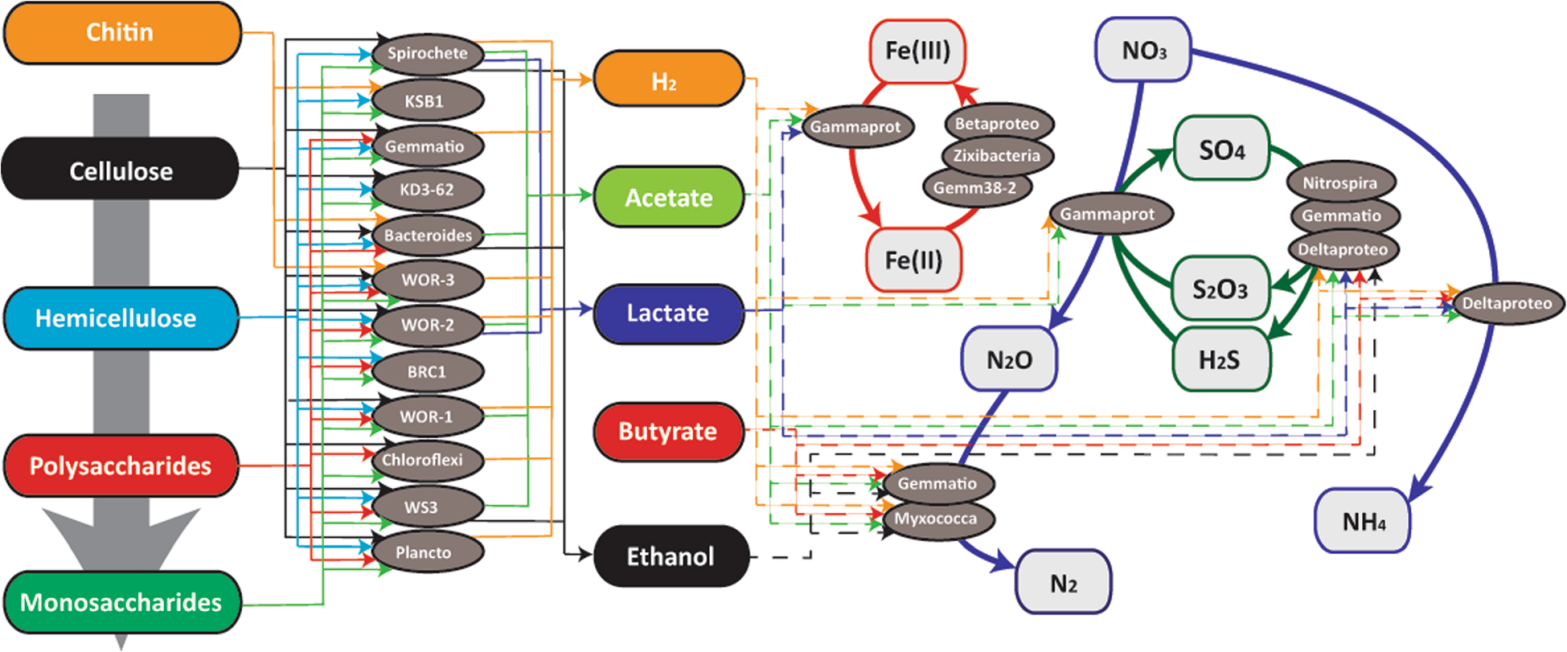
**Flow diagram of the potential interactions between (left to right) organic carbon utilization, fermentation, and respiration identified in the bacterial genomes reconstructed in this study.** Arrows represent metabolic capabilities that were identified in the metagenomic reconstruction from the White Oak River estuary. The dashed lines on the right represent potential electron donors for the anaerobic respiration processes. Note that the *Gammaproteobacteria* are capable of coupling nitrate reduction to either thiosulfate or sulfide oxidation. Abbreviations in the diagram are as follows; DNRA, dissimilatory nitrate reduction to ammonia; ‘Betaprot’, *Betaproteobacteria*; ‘Deltaprot’, *Deltaproteobacteria*; ‘Gemmatio’, *Gemmatimonadetes* (‘Gemm38-2’ refers specifically to bin 38-2), ‘Gammaprot’, *Gammaproteobacteria*; ‘Myxococca’, *Myxococcales*; ‘Plancto’, *Planctomycetes*.

Several of the bacterial groups capable of hydrolyzing complex organic carbon also have pathways for glycolytic fermentation of glucose to acetate, including WOR-1 (Figure 4), WOR-2, WS3, *Bacteroidetes*, *Nitrospira* (SG8-3), and *Spirochetes* (bin DG-61). All these groups have the reductive acetyl-CoA (Wood-Ljungdahl) and phosphate acetyltransferase-acetate kinase pathways for carbon fixation and acetate production; however, the WS3 and WOR-1 genomes appear to lack acetyl-CoA synthetase, which is essential to the Wood-Ljungdahl pathway. The *Spirochetes* and WOR-2 genomes contain lactate dehydrogenase genes suggesting they are also capable of lactate fermentation. The *Bacteroidetes*, *Spirochetes*, and *Thermodesulfovibrio*-like bins have genes that encode aldehyde dehydrogenase and alcohol dehydrogenase, suggesting they are capable of full fermentation to ethanol. Fermentation has been demonstrated in *Thermodesulfovibrio* spp. cultures [20]. The genome bins did not yield an identifiable complete pathway for butyrate formation. The end products of these fermentation pathways fuel terminal respiration in the sediment community; the key electron donors acetate and hydrogen are among the principal drivers of sulfate reduction [21] (Figure 4).

Several of the bacteria possess genes for Ni,Fe-hydrogenases, which can be involved in H_2_ production or consumption. These genes are ubiquitous in *Gemmatiomonas*, *Myxococcales*, *Delta*-, and *Gammaproteobacteria* (SG-11, SG-13, SG-15, SG-30, and SG-31) and are likely used for consumption of H_2_ by respiratory processes (for example, sulfate reduction and denitrification). The *Nitrospira* bin (SG8-35-4, related to *Thermodesulfovibrio* spp., Figure 1) contains genes for Ni,Fe-hydrogenases that are likely to participate in both sulfate reduction with hydrogen as the electron donor and the fermentative production of H_2_, which have been demonstrated in *Thermodesulfovibrio* spp. [20]. Several bacterial groups that are capable of organic carbon degradation and fermentation have Ni,Fe-hydrogenase genes, including *Planctomycetes*, *Spirochetes*, *Chloroflexi*, WOR-2, WOR-3, and WOR-1 (Figure 4). The extensive distribution of these hydrogenases among both fermenting and respiring bacteria indicates that H_2_ is a highly dynamic electron carrier produced and consumed by a wide range of sediment microbes, as it is in other anoxic environments [22,23]. No genes for Fe,Fe-hydrogenases were identified, which are thought to primarily produce H_2_ [24].

### Dissimilatory sulfur and nitrogen cycling

Aquatic sediments are characterized by redox gradients, as oxidized compounds are gradually reduced by respiration. To determine the respiratory repertoire of the bacterial community members, the estuary genomic bins were surveyed for key genes of respiration pathways. Several of the genomic bins contain dissimilatory sulfite reductase (*dsr*) genes, indicative of microbial sulfate and sulfite reduction. To account for the possibility that these genes belong to a phylogenetically distinct group of genes (rdsr) that mediate the reverse reaction [25], we generated a phylogenetic tree of all the *dsr*-like genes recovered (Additional file 1: Figure S3). The *Deltaproteobacteria*, which constituted the most abundantly detected microorganisms in the sulfate-rich zone (Additional file 1: Figure S2), have reducing-type *dsr* genes and complete sulfate reduction pathways (with the exception of the two *Myxococcales* bins). The bin SG8-35-4, a sister lineage to *Thermodesulfovibrio* spp. within the *Nitrospira* phylum, is capable of sulfate reduction based on the presence of *dsr* genes.

A complete sulfate reduction pathway was identified in the *Gemmatiomonas*-like bin SG8-17. The *dsrAB* genes from this bin fall within a phylogenetically deeply branched unknown clade (Additional file 1: Figure S3). This clade (designated DSR-J [26]) includes *dsrAB* sequences from an intertidal sand flat, Hydrate Ridge, deep-sea, and estuary sediments, suggesting these sulfate reducers are widespread in coastal and marine sediments. Genes of this clade were hypothesized to have been horizontally transferred [26]. No *dsr* genes were identified in any of the other *Gemmatiomonadetes*-like bins. Since all of the sequences for this clade had previously been recovered from large-insert (fosmid) clones that lacked 16S rRNA genes, their taxonomic affiliation had been uncertain. In this dataset the *dsrAB* genes are located on a 9.6 kb contig that is confidently binned, with both the tetranucleotide and coverage signatures, consistent with assignment to SG8-17. This genomic bin contains additional genes for sulfate reduction on other contigs, including *aprAB*, SAT, and *dsrC*. Thus, this member of the *Gemmatimonadetes* appears to be capable of sulfate reduction.

Up to 95% of the sulfide and thiosulfate generated by sulfate reduction is re-oxidized to sulfate in marine sediments [27]. Interestingly, the genomes of the most abundant *Gammaproteobacteria* (SG8-11, SG8-15, SG8-45, SG8-47 SG8-50, SMTZ1-46, and SMTZ-46) include genes for sulfur oxidation (*rdsr*, *apr*, and SAT) and pathways for thiosulfate oxidation (*soxABDZY* genes). Four bins contain sulfide quinone oxidoreductase genes (*sqr*) required for the oxidation of sulfide (SG8-15, SG8-30, SG8-47, and SG8-50). These *sqr*-containing *Gammaproteobacteria* are phylogenetically distinct and are related to *Thioalkalivibrio* sp. (Figure 1); unless relevant genomes have been missed, sulfide oxidation in the WOR sediments appears to be mediated by this group. All of these *Gammaproteobacteria* genomes contain genes for nitrate reduction.

**Figure 5:**
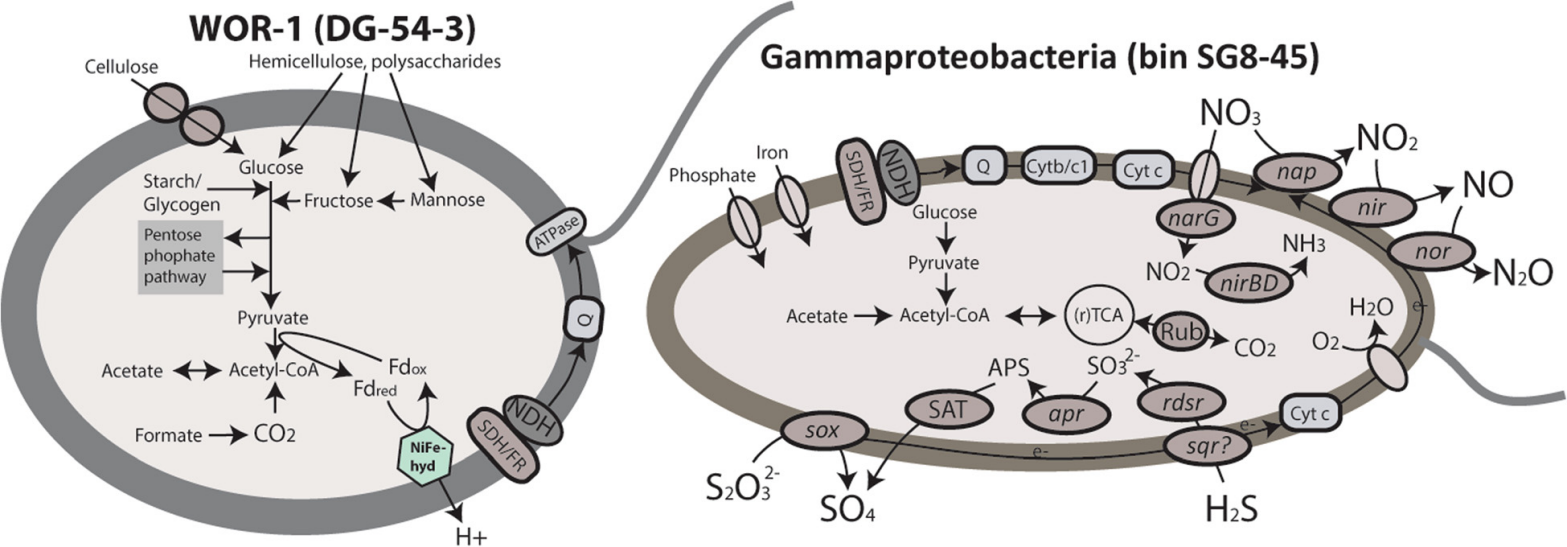
**Diagrams of metabolic potential and electron transport of WOR-1 (bin DG-54-3) and** ***Gammaproteobacteria*** **(bin SG8-45), based on gene content.** ATPase, ATP synthetase; FDH, formate dehydrogenase; NiFe-hyd, Ni,Fe-hydrogenase; Cytb/c1, quinone cytochrome oxidoreductase; Cyt c, cytochrome c; *nap*/*nar*, nitrate reductase; *nir*, nitrite reductase; *nor*, nitric oxide reductase; SAT, sulfate transferase; *apr*, APS reductase; *rdsr*, reverse dissimilatory sulfite reductase; Rub, RuBisCO; Q, quinine; SDH/FR, succinate dehydrogenase/fumarate reductase; NDH, NADH dehydrogenase; SOX, sulfur oxidation multienzyme complex. The sulfide quinone oxidoreductase (*sqr*) gene was not identified in this particular genomic bin, however many other closely related *Gammaproteobacteria* do have *sqr* genes.

Many bins within *Beta*- and *Gammaproteobacteria* (SG8-30, SG8-31, SG8-41, SG8-45, and SG8-46) also have *nirS* and *norBC* genes for the reduction of nitrite to nitrous oxide, N_2_O (Figure 5). However, genes for nitrous oxide reductase (*nosZ*) were only found in *Gemmatimonadetes* (SG8-23) and *Myxococcales* (SG-38 and -38-1) bins, suggesting they are reducing the N_2_O produced by *Gammaproteobacteria* to N_2_ (Figure 4). Two gammaproteobacterial genomes (SG8-30 and SG8-31) lack the sox pathway and *rdsr* genes, but genes for Ni-Fe-hydrogenases were present, suggesting they utilize H_2_ or organic carbon rather than sulfur species as an electron donor for nitrate reduction.

**Figure 6:**
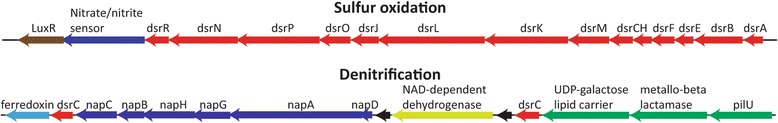
**Operons for sulfur oxidation and nitrate reduction present in the dominant** ***Gammaproteobacteria*** **genotypes.** Those shown here are present in the SG8-45 bin. However, syntenous operons are also present in several other *Gammaproteobacteria* bins (SG8-11, SG8-15, SG8-45, SG8-47, SG8-50, STMZ1-46, and SMTZ-46).

In several *Gammaproteobacteria*, the gene clusters for nitrate reduction and sulfur oxidation are mutually intertwined. The *rdsr* gene cluster of bins SG8-11, SG8-15, SG8-45, SG8-47, and SG8-50 includes a gene with homology to a nitrate sensor (*narX*) and a *luxR*-like transcription regulator (Figure 6). This type of sensor protein has been implicated in gene expression in response to changes in nitrate/nitrite concentrations [28]. Further, the gene cluster for nitrate reduction (*napABCDGH*) in the gammaproteobacterial bins SG8-45 and SG8-50 contains two *dsrC* genes, which have been suggested to regulate *rdsr* gene expression [29]. The presence of two *dsrC* genes in the *nap* operon suggests that regulation of sulfur oxidation and nitrate reduction is coordinated, consistent with the coupling of these processes in *Thioalkalivibrio* spp. [30,31], a close relative of these bins. However, since the functions of *dsrC* and *narX* genes are putative, this inference needs to be verified with experimental evidence. The enrichment of these *Gammaproteobacteria* in shallow sediment samples, where the sulfide porewater concentrations decrease to the detection limit [32], is consistent with nitrate-dependent oxidation of sulfur compounds. Genes homologous to cytochrome c oxidases are present in SG8-11, SG8-31, SG8-45, and SG8-50, suggesting that some of the *Gammaproteobacteria* are also capable of O_2_ reduction.

Dissimilatory nitrate reduction to ammonia (DNRA) has been shown to co-occur with denitrification in estuary sediments [33]. To look for the presence of DNRA genes in the genomes, we constructed a phylogenetic tree of all the formate-dependent nitrite reductase (*nrfA)* genes identified in genomic bins (Additional file 1: Figure S4). Most (72%) of these do not fall into a clade that has been described as true *nrfA* genes [34,35]. Two *Deltaproteobacteria* bins (SG8-35 and SG8-35-2) were confidently identified as being involved in DNRA.

### Iron cycling

While the microbial cycling of iron in marine sediments has been commonly documented by geochemical approaches [36], well-documented biochemical pathways for iron oxidation and reduction now enable environmental genetic studies of these processes as well. For example, periplasmic and outer-membrane-anchored c-type cytochromes and a beta-barrel protein within the metal reduction (Mtr) respiratory pathway are essential for respiratory electron transport across the outer cell membrane to iron minerals in *Shewanella oneidensis* [37,38]. Homologues of these enzymes catalyze lithotrophic iron oxidation in *Sideroxydans lithotrophicus* [39] and phototrophic iron oxidation in *Rhodopseudomonas palustris* [40]. Genes with homology to *mtr* genes are present in seven different WOR genomic bins. Betaproteobacterial genomes reconstructed from the sulfate-rich zone (SG8-39, SG8-40, and SG8-41) have *mtrABC* genes with sequence identity (40% to 50%, 23% to 35%, and 26% to 28% at the protein level, respectively) to those found in *Shewanella* and *Geobacter* spp., suggesting that they are capable of iron reduction. *Gemmatimonadetes* bin SG8-38-2 has a putative *mtrABC* gene cluster and may be involved in iron cycling as well. Consistent with Zixibacteria genomes obtained from groundwater, bin SM-73-2 contains *mtrAB* but lacks the genes for extracellular cytochromes implicated in iron reduction [37]. The *Gammaproteobacteria* bin SG8-47 contains both the genes homologous to *mtrABC* and a cytoplasmic membrane-associated c-type cytochrome (*cymA*) required for iron reduction [41]. The closely related bacteria SG8-11 and SG8-30 have all these genes except *mtrC* and *cymA*, respectively. Bacteria potentially capable of iron reduction have multiple multi-heme cytochromes [42]; *Gammaproteobacteria* bins SG8-11 and SG8-30 also each have genes encoding six unique types of these cytochromes.

## Conclusions

The highly resolved genomic reconstruction of estuary sediment microbial populations revealed potential physiological pathways of individual community members, including several recently defined (for example, KD3-62, TA06, Zixibacteria, and BRC1) and three newly described (WOR-1, WOR-2, and WOR-3) uncultured candidate phyla. The 12 genome bins belonging to WOR-1, WOR-2, WOR-3, KD3-62, and TA06 are the first to be constructed from these phyla. Based on their genome sequences, several of these groups appear to be capable of hydrolysis and fermentation of a variety of organic compounds, expanding the range of bacterial phyla known to hydrolyze and ferment biopolymers (sugars and proteins) to low molecular weight substrates. An average of 14 carbohydrate hydrolases was found per genome, with WOR-1 and WOR-2 having the most with 20 and 39, respectively. *Planctomycetes* and *Bacteroides* are also among the most versatile carbohydrate degrading bacteria in the White Oak River sediments with an average of 30 and 80 hydrolytic genes per genome, respectively.

This study identified potential new bacterial capabilities in sulfur cycling. One uncultured *Gemmatimonadetes* bacterium was linked to a previously taxonomically unassigned *dsr* gene clade, suggesting that this group is capable of sulfate reduction, a process that is commonly catalyzed by *Deltaproteobacteria*. Sequences for this group have been recovered from sediments throughout the world, suggesting a cosmopolitan *Gemmatimonadetes* lineage of sulfate-reducing bacteria. The oxidation of sulfide and thiosulfate is essential to sulfur cycling in marine and coastal sediments [43]; the ongoing microbial census of these processes is uncovering new key populations, such as uncultured *Gammaproteobacteria* mediating sulfur oxidation in coastal sediments [43]. Several of the most abundant *Gammaproteobacteria* in the sulfate-rich zone described here have the genetic potential for coupling anaerobic sulfur or hydrogen oxidation to reduction of nitrate to nitrous oxide, as documented in *Beggiatoa* spp. in marine sediments as well [44]. Interestingly, the genomic potential for the next denitrification step (N_2_O to N_2_) in the White Oak River estuary sediments is found in different organisms, the *Gemmatiomadetes* and *Myxococcales*. This apparent splitting of the denitrification pathway over multiple taxa, and concurrent leakage of the intermediate N_2_O, provide an explanation as to why estuaries function as a significant source of N_2_O to the atmosphere [45].

Placing potential metabolic capabilities of individual populations within the framework of other community members provided a wiring diagram of the potential biogeochemical interactions at a system level. This study demonstrates how key pathways of carbon degradation and sulfur, nitrogen, and iron cycling may be distributed over a previously unexplored range of bacterial phyla within the estuarine sediment community; the genomic analysis provides a new perspective on the functioning of these pathways in nature, characterized by a high degree of functional redundancy among different lineages and by metabolic plasticity within specific organisms [46]. The comprehensive genomic reconstruction of these sediment communities provides a wide spectrum of specific links between metabolic potential and diversity and reveals potential ecological interactions within the communities; it begins to chart the possible flow of carbon and nutrients through the multiple layers of microbial processing, assimilation, and remineralization in the estuarine environment.

## Methods

### Sample collection and processing

Six 1-m plunger cores were collected from approximately 1.5-m water depth in three mid-estuary locations (two cores per site) of the White Oak River, North Carolina in October 2010 (site 1 at 34°44.592 N, 77°07.435 W; site 2 at 34°44.482 N, 77°07.404 W, and site 3 at 34°44.141 N, 77°07298 W). Cores were stored at 4°C overnight and processed 24 h after sampling, as detailed in Lazar *et al.* [32]. Each core was sectioned into 2-cm intervals. From each site, one core was subsampled for geochemical analyses and the others were subsampled for DNA extractions. DNA was extracted using the UltraClean Mega Soil DNA Isolation Kit (MoBio, Carlsbad, CA, USA), using 6 g of sediment, and stored at −80°C until use. Sulfate, sulfide, and methane profiles of these cores at the three sites can be seen in Lazar *et al.* [32]. The SMTZ is located at around 26, 24, and 16 cm in sites 1, 2, and 3 (respectively); therefore, the samples above that depth represent the sulfate-rich zones and those below the methane-rich zones.

### Genomic assembly, binning, and annotation

Illumina (HiSeq 2000 PE100) shotgun genomic reads were screened against Illumina artifacts (adapters, DNA spike-ins) (Illumina Inc., San Diego, CA, USA) with a sliding window kmer size of 28 and a step size of 1. Reads with 3 or more N’s or with average quality score of less than Q20 and a length <50 bps were removed. Screened reads were trimmed from both ends using a minimum quality cutoff of 5 using Sickle (https, //github.com/najoshi/sickle). Trimmed, screened, paired-end Illumina reads were assembled using IDBA-UD [47] with the following parameters (--pre_correction --mink 55 --maxk 95 --step 10 --seed_kmer 55). To maximize assembly, reads from different sites were co-assembled.

The shallow assembly was a combination of high-quality reads (474,179,948 with an average read length 148 bp) from sites 2 (8 to 12 cm) and 3 (8 to 10 cm). The SMTZ assembly was generated from a combination of high-quality reads (698,574,240, average read length 143 bp and average insert 274 bp) from sites 2 (30 to 32 cm) and 3 (24 to 28 cm). The deep assembly was generated from high-quality reads (378,027,948, average read length 124 bp and average insert 284 bp) of site 1 (52 to 54 cm). Since we were not able to co-assemble all three of the samples from the SMTZ due to computational limits, an additional assembly was generated from the third sample (site 1, 26 to 30 cm) from high-quality 345,710,832 reads (average length of 129 bp and average insert 281 bp). The contigs from this sample were co-binned with the assembly of the other two samples (sites 2 and 3, described above). This resulted in some closely related but unique bins, for example, the *Gammaproteobacteria* bins SMTZ1-46 (from site 1) and SMTZ-46 (from the site 2 and 3 assembly). Contigs with genes of particular interest were checked for chimeras by looking for dips in coverage within read mappings.

Initial binning of the assembled fragments was done using tetra-nucleotide frequencies signatures using 5 kb fragments of the contigs, as detailed in Dick *et al.* [48]. ESOM maps were manually delineated and curated based on clusters within the map (as shown in Additional file 1: Figure S1). This binning was enhanced by incorporating coverage signatures for all of the assembled contigs into the ESOM maps [49,50]. Coverage was determined by recruiting reads (from each individual library/sample) to scaffolds by BLASTN (bitscore >75) and then normalized to the number of reads from each library, determining which genomic bin each of the 5 kb sub-portions of the contigs were assigned then assessed the accuracy of the binning. Contigs larger than 15 kb were assigned to the bin where the majority of the 5 kb sub-portions were assigned. The completeness of the genomes within bins was then estimated by counting single-copy genes using CheckM [51]. Some of these bins were then shown to contain multiple (2-5) closely related genomes based on the presence of multiple copies of single-copy genes. Those bins were further separated by plotting differential coverage of scaffolds between two libraries. Distinct clusters of scaffolds on the coverage plots were manually delineated into new bins. Binning was also manually curated based on GC content, top blast hits, and mate-pairings.

Genes were called and putative function was assigned using the JGI IMG/MER system [52]. The functions of predicted genes (including all those discussed in detail here) were manually curated and revised by comparison of homology to a variety of databases including KEGG, pfam, NCBI, and COG. The CAZy database was used to identify carbohydrate active genes [16]; a subset was selected which has been shown to be involved in specific carbohydrate degradation pathways (Additional file 3: Table S6) [22,53].

### Phylogenetic analyses

The concatenated ribosomal protein tree was generated using syntenic genes that have been shown to have undergone limited lateral gene transfer (rpL2, 3, 4, 5, 6, 14, 15, 16, 18, 22, 24 and rpS3, 8, 10, 17, 19) [54]. The reference data sets were derived from the Phylosift database [55], with additional sets from the Joint Genomic Institute IMG database as detailed in Castelle *et al.* [56]. Scaffolds containing <50% of these 16 genes were not included in the analyses. Only two bins (DG-61 and DG-78) remained below this cutoff. We searched NCBI to include additional closely related reference sequences. Amino acid alignments of the individual genes were generated using MUSCLE [57] and manually curated. The curated alignments were then concatenated for phylogenetic analyses. The ribosomal protein tree included 182 taxa and 2,411 unambiguously aligned positions. The phylogeny shown in all the figures were generated using maximum likelihood using Phyml [58]. Bootstrap values were generated from 100 replicates of UPGMA tree building method and Jukes-Cantor distance modeling.

### Availability of supporting data

The full metagenomic assemblies presented in this study are available in IMG with the following IMG Taxon IDs. They are Taxon ID: 3300001855 (sulfate-rich assembly, ‘SG’ genomes), 3300002052 (SMTZ site 1 assembly, ‘SM1’ genomes), 3300001753 (SMTZ sites 2 and 3 assembly, ‘SM23’ genomes), and 3300001854 (methane-rich assembly, ‘DG’ genomes). The genomic bins supporting the results of this article are being made available in NCBI Genbank under the BioProjectID PRJNA270657.

## Additional files

Additional file 1:
**Supplementary data Table S1.** General characteristics of the large fraction (contigs larger than 5 kb) of the four assemblies generated and used for binning, in the study. **Table S2.** Summary of genome reconstruction completeness, contamination, and strain heterogeneity based on CheckM package [51]. For more detailed information about individual genomic bins, see Table S3. **Table S3.** Number of phylogenetic proteins as identified using Phylosift in each of the genomic bins. **Table S4.** General characteristics of all bacterial genomic bins. **Figure S1.** Tetra-nucleotide ESOM binning map of shallow assembly. Each data point is a 5-kb portion of DNA sequence. Separation of data points are visible as brown lines (background color) which delineates clustering of sequences by the mapping. The manually delineated bins are colored and labeled. Notice that in a few cases (SG8-35, SG8-35-1, SG8-35-2, and SG8-38 and 38-1), multiple closely-related bins fall within one large cluster on this map. These bins were found to contain more than one genome and were therefore further delineated based on differential coverage plots. **Figure S2.** Abundances of top genotypes in the SMTZ (24 to 32 cm) and methane-rich (44 to 48 cm) layers of the sediment profiles based on the number of reads that map to all the genes for ribosomal protein S3. Those that are represented in the genomic bins are labeled. **Figure S3.** Phylogenetic tree of concatenated *dsrAB* genes within bacteria genomic bins from this study. Closed and open circles represent maximum likelihood (ProML, ARB package) bootstrap value >75 and >50, respectively. **Figure S4.** Phylogenetic tree of WOR proteins annotated at formate-dependent nitrite-reductase (periplasmic cyctochrome c552), NrfA. Cluster of sequences considered to be involved in DNRA are delineated on the right (Welsh *et al*. [34]). Genomic bin designations are shown in bold. Additional file 2: Table S5. Table showing the abundance and identification of genes involved in protein degradation in the genomic bins in this study. The pfam and EC numbers used to search the annotations are provided. The number in each cell represents the number of that particular gene family that was identified. Additional file 3: Table S6. A complete list of the abundance and specific genes in the genomic bins involved in degradation of organic carbon compounds identified by comparison to the CAZy database. The number represents the number of that particular gene that was found in that bin.
