# Supplementary material for: Genomic resolution of linkages in carbon, nitrogen, and sulfur cycling among widespread estuary sediment bacteria
Source: Microbiome. 2015 Apr 13;3:14. doi: 10.1186/s40168-015-0077-6 (PMC4411801; doi:10.1186/s40168-015-0077-6)
Supplement: Additional file 1: — Supplementary data Table S1. General characteristics of the large fraction (contigs larger than 5 kb) of the four assemblies generated and used for binning, in the study. Table S2. Summary of genome reconstruction completeness, contamination, and strain heterogeneity based on CheckM package [51]. For more detailed information about individual genomic bins, see Table S3. Table S3. Number of phylogenetic proteins as identified using Phylosift in each of the genomic bins. Table S4. General characteristics of all bacterial genomic bins. Figure S1. Tetra-nucleotide ESOM binning map of shallow assembly. Each data point is a 5-kb portion of DNA sequence. Separation of data points are visible as brown lines (background color) which delineates clustering of sequences by the mapping. The manually delineated bins are colored and labeled. Notice that in a few cases (SG8-35, SG8-35-1, SG8-35-2, and SG8-38 and 38-1), multiple closely-related bins fall within one large cluster on this map. These bins were found to contain more than one genome and were therefore further delineated based on differential coverage plots. Figure S2. Abundances of top genotypes in the SMTZ (24 to 32 cm) and methane-rich (44 to 48 cm) layers of the sediment profiles based on the number of reads that map to all the genes for ribosomal protein S3. Those that are represented in the genomic bins are labeled. Figure S3. Phylogenetic tree of concatenated dsrAB genes within bacteria genomic bins from this study. Closed and open circles represent maximum likelihood (ProML, ARB package) bootstrap value >75 and >50, respectively. Figure S4. Phylogenetic tree of WOR proteins annotated at formate-dependent nitrite-reductase (periplasmic cyctochrome c552), NrfA. Cluster of sequences considered to be involved in DNRA are delineated on the right (Welsh et al. [34]). Genomic bin designations are shown in bold. [file 40168_2015_77_MOESM1_ESM.pdf]

Supplementary information for

Genomic resolution of linkages in carbon, nitrogen, and sulfur cycling among widespread estuary sediment bacteria

Brett J. Baker[1*], Cassandre Lazar[2], Andreas Teske[2], and Gregory J. Dick[1,3]
*email acidophile@gmail.com. To whom correspondence should be addressed.

Table S1. General characteristics of the large fraction (contigs larger than 5-kb) of the four assemblies generated, and used for binning, in the study.

**Assembly**	**Sulfate-rich (sites 2 and 3)**	**SMTZ (sites 2 and 3)**	**SMTZ (site 1)**	**Methane-rich (site 1)**
**Total length (bp)**	366,573,198	799,007,063	406,438,721	449,385,852
**Total number of contigs**	34,334	72,542	38,359	44,276
**Largest contig (bp)**	457,137	549,755	332,158	277,131
**Average contig size**	16,805	19,846	16,317	16,249
**N50**	11,108	11,897	11,245	10,464
**Number of predicted ORFs**	401,780	901,653	462,000	514,942
**Number of ORFs with functional prediction**	276,967 (69%)	610,634 (68%)	312,636 (68%)	344,833 (67%)

Table S2. Summary of genome reconstruction completeness, contamination, and strain heterogeneity based on CheckM package [51]. For more detailed information about individual genomic bins see Table S3.

Bin Id	Taxonomy	# markers	# marker sets	0	1	2	3	4	5+	Completeness	Contamination	Strain heterogeneity
SM23-62-1	*Bacteroides*	317	211	28	262	25	1	1	0	89.3	6.52	29.41
SM23-62	*Bacteroides*	317	211	31	273	12	1	0	0	87.89	5.13	13.33
SM1-62	*Bacteroides*	279	187	127	149	3	0	0	0	54.7	1.07	0
SG8-41	*Betaproteobacteria*	421	212	28	366	26	1	0	0	91.16	5.31	3.45
SG8-40	*Betaproteobacteria*	421	212	109	302	10	0	0	0	73.7	2.36	20
SG8-39	*Betaproteobacteria*	422	212	66	347	7	2	0	0	81.44	1.56	7.69
SM23-51	BRC1	148	92	54	92	1	0	1	0	53.01	4.35	0
SM23-84	*Chloroflexi;Anaerolineae*	160	109	34	102	17	6	1	0	76.45	23.85	7.32
SM23-63	*Chloroflexi;Anaerolineae*	163	110	16	121	26	0	0	0	87.73	18.64	38.46
SG8-19	*Chloroflexi;Anaerolineae*	163	110	45	109	8	0	1	0	73.56	8.48	7.14
SM23-28-2	*Chloroflexi;Dehalococcoidia*	151	101	33	118	0	0	0	0	71.29	0	0
SM23-28-1	*Chloroflexi;Dehalococcoidia*	151	101	98	52	1	0	0	0	26.29	0.99	100
SG8-51-3	*Chloroflexi;Dehalococcoidia*	103	57	35	55	13	0	0	0	49.84	3.51	0
DG-22	*Chloroflexi;Dehalococcoidia*	151	101	74	76	1	0	0	0	46.74	0.99	0
DG-18	*Chloroflexi;Dehalococcoidia*	151	101	84	61	6	0	0	0	38.12	2.74	33.33
SM23-39	*Chlymidae*	231	149	30	200	1	0	0	0	82.89	0.67	0
SM23-61	*Deltaproteobacteria*	248	156	35	202	10	1	0	0	85.75	7.37	7.69
SG8-13	*Deltaproteobacteria*	248	156	28	215	5	0	0	0	84.94	1.92	20
DG-28	*Deltaproteobacteria*	248	156	138	104	6	0	0	0	38.73	1.6	16.67
SG8-35-2	*Deltaproteobacteria; Desulfobacterales*	281	168	17	226	33	5	0	0	91.22	16.98	25
SG8-35	*Deltaproteobacteria; Desulfobacterales*	281	168	49	201	25	6	0	0	81.46	14.34	18.6
DG-60	*Deltaproteobacteria;Syntrophobacter*	281	168	86	180	15	0	0	0	63.12	0.33	0
DG-40	*Gammaproteobaciteria/Coxiella*	271	173	48	194	29	0	0	0	77.36	8.04	0
SG8-47	*Gammaproteobacteria*	104	58	11	76	17	0	0	0	82.76	6.9	29.41
SG8-31	*Gammaproteobacteria*	104	58	0	9	89	6	0	0	100	99.69	53.27
SG8-30	*Gammaproteobacteria*	483	277	96	371	14	2	0	0	75.13	3.66	0
SG8-15	*Gammaproteobacteria*	546	285	183	336	27	0	0	0	61.61	5.34	11.11
SG8-11	*Gammaproteobacteria*	546	285	242	292	12	0	0	0	55.71	2.32	0
SM23-46	*Gammaproteobacteria;Acidithiobacillales*	262	165	53	199	10	0	0	0	79.55	4.28	30
SM1-46	*Gammaproteobacteria;Acidithiobacillales*	262	165	24	231	7	0	0	0	92.63	3.13	14.29
SG8-45	*Gammaproteobacteria;Acidithiobacillales*	262	165	35	202	25	0	0	0	84.75	10.24	32
SG8-50	*Gammaproteobacteria;Thiothrichales*	546	285	220	209	101	15	1	0	56.55	21.47	1.97
SM23-52	*Gemmatiomonas*	148	92	15	110	22	1	0	0	91.64	22.83	60
SG8-38-2	*Gemmatiomonas*	148	92	62	78	8	0	0	0	48.87	5.5	0
SG8-28	*Gemmatiomonas*	148	92	34	107	7	0	0	0	76.12	6.62	0
SG8-23	*Gemmatiomonas*	104	58	15	70	17	2	0	0	83.7	8.93	4.35
SG8-17	*Gemmatiomonas*	148	92	50	78	17	3	0	0	60.13	12.84	26.92
DG-56	KD3-62	161	108	71	89	1	0	0	0	51.23	0.93	0
SM23-57	KSB1	148	92	82	57	9	0	0	0	42.16	7.19	0
SM23-31	KSB1	148	92	47	100	1	0	0	0	59.01	1.09	100
SG8-38-1	*Myxococcales*	103	57	10	71	22	0	0	0	89.47	14.04	72.73
SG8-38	*Myxococcales*	248	156	32	211	4	1	0	0	81.09	3.23	28.57
SG8-3	*Nitrospira*	248	156	53	113	63	15	4	0	83.77	53.74	8.33
SM23-35	*Nitrospira/ like Thermodesulfovibrio*	182	111	55	117	10	0	0	0	65.97	5.46	0
SG8-35-4	*Nitrospira/ like Thermodesulfovibrio*	182	111	54	111	15	2	0	0	72.13	12.3	47.62
SG8-35-1	*Nitrospira/ like Thermodesulfovibrio*	182	111	32	107	40	3	0	0	80.59	31.22	2.04
SG8-24	OD1	151	101	36	113	2	0	0	0	69.11	1.98	50
DG-74-3	OD1	151	101	43	106	2	0	0	0	62.58	0.99	0
DG-74-2	OD1	104	58	15	38	47	4	0	0	76.85	43.31	0
DG-74-1	OD1	161	108	60	101	0	0	0	0	53.52	0	0
DG-72	OD1	104	58	15	85	2	2	0	0	77.87	5.75	0
DG-75	OP11	160	109	86	72	2	0	0	0	34.71	0.54	100
SM23-65	*Planctomyce*	144	89	55	81	8	0	0	0	49.07	2.2	0
SM23-32	*Planctomyce*	144	89	36	106	2	0	0	0	63.48	2.25	0
SM23-25	*Planctomyce*	144	89	40	99	5	0	0	0	62.08	4.49	0
DG-58	*Plantomycetes*	144	89	53	89	2	0	0	0	47.12	1.12	0
DG-23	*Plantomycetes*	144	89	15	126	3	0	0	0	83.15	2.31	33.33
DG-20	*Plantomycetes*	144	89	45	87	12	0	0	0	57.87	4.19	0
SM23-33	*Plantomycetes/Phycisphaerae*	144	89	25	118	1	0	0	0	75.28	1.12	0
SM23-32-1	*Plantomycetes/Phycisphaerae*	144	89	55	75	13	1	0	0	44.94	5.99	18.75
SM23-30	*Plantomycetes/Phycisphaerae*	144	89	30	114	0	0	0	0	71.15	0	0
SM1-79	*Plantomycetes/Phycisphaerae*	144	89	56	83	4	1	0	0	49.34	6.74	0
SG8-4	*Plantomycetes/Phycisphaerae*	144	89	29	114	1	0	0	0	71.91	0.56	0
DG-61	*Spirochete*	216	126	29	163	18	6	0	0	89.61	22.49	8.33
SM23-40	TA06	148	92	7	141	0	0	0	0	92.39	0	0
SM1-40	TA06	148	92	14	133	1	0	0	0	84.78	1.09	100
DG-78	TA06	144	90	39	103	2	0	0	0	75.88	1.18	0
DG-26	TA06	148	92	18	116	14	0	0	0	83.15	7.99	7.14
DG-24	TA06	148	92	39	109	0	0	0	0	64.13	0	0
DG-54-3	WOR-1	103	58	14	50	36	2	1	0	78.45	38.79	0
SM23-72	WOR-2	104	58	6	16	79	3	0	0	89.66	79.44	70.45
SM23-29	WOR-2	153	94	14	79	48	12	0	0	89.64	57.98	16.67
SM23-60	WOR-3	144	90	9	110	24	1	0	0	90.56	23.89	25.93
SM23-42	WOR-3	148	92	6	141	1	0	0	0	93.48	1.09	0
SM1-77	WOR-3	144	90	103	41	0	0	0	0	26.88	0	0
DG-63	WS3	148	92	5	125	18	0	0	0	95.59	16.3	0
SM23-81	Zixibacteria	148	92	6	138	4	0	0	0	94.5	4.35	0
SM23-73-3	Zixibacteria	148	92	29	119	0	0	0	0	73.72	0	0
SM23-73-2	Zixibacteria	148	92	21	124	3	0	0	0	86.2	3.26	33.33
SM23-73	Zixibacteria	148	92	51	80	16	1	0	0	54.28	13.88	5.26
SM1-73	Zixibacteria	148	92	27	117	4	0	0	0	77.83	3.32	25
DG-27	Zixibacteria	148	92	46	102	0	0	0	0	55.73	0	0

Table S3. Number of phylogenetic proteins as identified using Phylosift in each of the genomic bins.

	ribosomal protein S2 rpsB (Archaea: 100%, Bacteria: 99.5%)	ribosomal protein S10 rpsJ (Archaea: 100%, Bacteria: 98.51%)	ribosomal protein L1 rplA (Archaea: 100%, Bacteria: 99.83%)	translation initiation factor IF-2 (Archaea: 100%, Bacteria: 99.83%)	metalloendopeptidase (Archaea: 100%, Bacteria: 99.83%)	ribosomal protein L22 (Archaea: 100%, Bacteria: 99.67%)	ribosomal protein L4/L1e rplD (Archaea: 100%, Bacteria: 99.67%)	ribosomal protein L2 rplB (Archaea: 100%, Bacteria: 99.5%)	ribosomal protein S9 rpsI (Archaea: 100%, Bacteria: 100%)	ribosomal protein L3 rplC (Archaea: 100%, Bacteria: 99.5%)	phenylalanyl-tRNA synthetase beta subunit (Archaea: 100%, Bacteria: 99.67%)	ribosomal protein L14b/L23e rplN (Archaea: 100%, Bacteria: 99.34%)	ribosomal protein S5 (Archaea: 100%, Bacteria: 99.5%)	ribosomal protein S19 rpsS (Archaea: 100%, Bacteria: 99.17%)	ribosomal protein S7 (Archaea: 100%, Bacteria: 99.67%)	ribosomal protein L16/L10E rplP (Archaea: 100%, Bacteria: 99.67%)	ribosomal protein S13 rpsM (Archaea: 100%, Bacteria: 99.17%)	phenylalanyl-tRNA synthetase alpha subunit (Archaea: 100%, Bacteria: 99.83%)	ribosomal protein L15 (Archaea: 100%, Bacteria: 99.5%)	ribosomal protein L25/L23 (Archaea: 100%, Bacteria: 99.17%)	ribosomal protein L6 rplF (Archaea: 100%, Bacteria: 99.5%)	ribosomal protein L11 rplK (Archaea: 100%, Bacteria: 99.83%)	ribosomal protein L5 rplE (Archaea: 100%, Bacteria: 99.83%)	ribosomal protein S12/S23 (Archaea: 100%, Bacteria: 99.17%)	ribosomal protein L29 (Archaea: 98.39%, Bacteria: 98.68%)	ribosomal protein S3 rpsC (Archaea: 100%, Bacteria: 99.83%)	ribosomal protein S11 rpsK (Archaea: 100%, Bacteria: 99.17%)	ribosomal protein L10 (Archaea: 98.39%, Bacteria: 99.67%)	ribosomal protein S8 (Archaea: 100%, Bacteria: 99.5%)	tRNA pseudouridine synthase B (Archaea: 95.16%, Bacteria: 97.35%)	ribosomal protein L18P/L5E (Archaea: 100%, Bacteria: 99.83%)	ribosomal protein S15P/S13e (Archaea: 100%, Bacteria: 99.84%)	Porphobilinogen deaminase (Archaea: 85.48%, Bacteria: 86.59%)	ribosomal protein S17 (Archaea: 100%, Bacteria: 99.17%)	ribosomal protein L13 rplM (Archaea: 100%, Bacteria: 99.83%)	ribonuclease HII (Archaea: 100%, Bacteria: 98.51%)	ribosomal protein L24 (Archaea: 100%, Bacteria: 99.5%)	
**SM23-62**	1	1	1	2	1	1	1	1	1	1	1	1	1	1	1	1	1	1	2	1	1	1	1	1	1	2	1	1	1	1	1	1	0	1	1	1	1	**Bacteroides**
**SM23-62-1**	1	1	1	1	2	1	1	1	1	1	1	1	2	1	1	1	2	1	2	1	2	1	1	1	1	1	2	1	2	1	2	1	0	1	1	1	2	**Bacteroides**
**SM1-62**	1	1	1	1	2	1	1	1	1	1	1	1	2	1	1	1	2	1	2	1	2	1	1	1	1	1	2	1	2	1	2	1	0	1	1	1	2	**Bacteroides**
**SG8-39**	1	1	1	4	2	1	1	1	1	1	1	1	1	1	1	1	1	1	1	1	1	1	1	1	1	1	1	1	1	0	2	0	1	1	1	1	1	**Betaproteobacteria**
**SG8-41**	1	1	1	2	1	1	1	1	1	1	0	1	1	1	2	1	1	0	1	1	1	1	1	2	1	1	1	1	1	1	1	1	1	1	1	1	1	**Betaproteobacteria**
**SG8-40**	1	1	1	2	1	1	1	1	1	1	1	1	0	1	1	1	0	1	0	1	0	1	1	1	1	1	1	1	1	1	0	0	1	1	1	0	1	**Betaproteobacteria**
**SM23-51**	1	1	1	0	0	1	1	2	0	1	1	1	1	1	0	1	1	0	1	1	1	1	1	0	1	1	1	1	1	0	1	1	0	1	0	0	1	**BRC1**
**SM23-31**	0	1	1	2	2	1	1	1	0	1	1	1	1	1	1	1	1	1	1	1	1	1	1	1	1	0	1	1	1	1	1	1	0	1	0	1	1	**KSB1**
**SG8-19**	2	0	1	3	1	0	0	0	1	0	1	1	1	0	1	1	1	1	1	0	1	1	1	1	1	1	1	1	1	0	1	1	0	1	1	1	1	**Chloroflexi;Anaerolineae**
**SM23-63**	1	1	1	2	2	1	1	1	1	1	1	1	1	1	1	1	1	1	1	1	1	1	1	1	1	1	1	1	1	1	1	1	1	1	1	2	1	**Chloroflexi;Anaerolineae**
**SM23-84**	2	1	1	3	2	1	1	1	1	1	2	1	1	1	0	1	1	1	1	1	1	1	1	0	1	1	1	1	1	2	2	0	0	1	1	1	1	**Chloroflexi;Anaerolineae**
**SG8-51-3**	0	1	1	0	0	2	1	2	0	1	1	2	1	2	2	2	1	1	1	1	1	1	1	2	2	2	1	1	2	0	1	1	0	2	0	1	2	**Chloroflexi;Dehalococcoidia**
**DG-18**	1	1	0	1	0	1	1	1	1	1	0	0	1	1	1	0	2	0	2	1	0	0	0	1	0	1	2	0	0	1	0	0	0	0	1	1	0	**Chloroflexi;Dehalococcoidia**
**DG-22**	1	0	0	0	1	1	1	1	0	0	1	1	1	1	0	1	1	1	1	1	1	0	1	1	1	2	1	0	1	0	1	1	1	1	0	1	1	**Chloroflexi;Dehalococcoidia**
**SM23-28-2**	1	1	1	1	1	1	1	1	1	1	1	1	1	1	1	1	1	1	1	1	1	1	1	1	1	1	1	1	1	1	1	1	1	1	1	1	1	**Chloroflexi;Dehalococcoidia**
**SM23-28-1**	1	1	0	0	0	1	1	1	0	1	0	1	1	1	0	1	0	0	1	1	1	0	1	0	1	1	0	0	1	0	1	0	1	1	0	0	1	**Chloroflexi;Dehalococcoidia**
**SM23-39**	1	1	1	2	1	1	1	1	1	1	1	1	1	1	1	1	1	1	1	1	1	1	1	1	1	1	1	1	1	1	1	1	0	1	1	2	1	**Chlymidae**
**DG-28**	0	1	2	0	0	1	1	1	1	1	1	1	1	1	0	1	0	1	1	1	1	2	1	0	1	1	0	2	1	0	1	0	0	1	1	0	1	**Deltaproteobacteria**
**SM23-72**	2	1	2	2	3	1	1	1	2	1	4	2	2	1	2	2	2	2	2	1	2	2	2	2	2	1	2	2	2	0	2	2	0	2	2	0	2	**WOR-2**
**SM23-29**	1	1	1	3	1	1	1	1	1	1	4	1	0	1	2	1	2	3	0	1	1	1	1	2	1	1	2	1	1	3	1	2	0	1	1	1	1	**WOR-2**
**SM23-61**	1	1	1	3	1	1	1	1	1	1	0	1	1	1	1	1	1	0	1	1	1	1	1	1	1	1	1	1	1	1	1	0	1	1	1	1	1	**Deltaproteobacteria**
**SG8-35-2**	1	1	1	5	1	1	1	1	2	1	1	1	1	1	1	1	1	1	1	1	1	1	1	1	1	1	1	1	1	1	1	1	1	1	2	1	1	**Deltaproteobacteria**
**SG8-35**	1	0	0	5	0	1	0	1	1	0	1	1	1	1	0	1	1	1	1	0	1	0	1	0	1	1	1	0	1	1	1	1	1	1	1	1	1	**Deltaproteobacteria**
**SG8-13**	1	1	1	1	1	1	1	1	1	1	1	1	1	1	1	1	1	1	1	1	1	1	1	1	1	1	1	1	1	1	1	1	1	1	1	1	1	**Deltaproteobacteria**
**DG-60**	1	0	1	2	1	1	0	0	1	0	1	2	2	1	1	1	2	0	2	0	2	1	2	1	1	1	2	1	2	1	2	1	0	1	1	1	2	**Deltaproteobacteria**
**DG-40**	0	1	1	2	1	1	1	1	1	1	0	1	2	1	1	1	1	0	2	1	2	1	2	1	1	1	1	1	2	1	1	1	0	1	1	1	2	**Gammaproteobaciteria/Coxiella**
**SG8-31**	2	1	1	3	2	2	1	2	2	1	3	2	2	2	2	2	2	3	2	2	2	1	2	2	2	2	2	1	2	2	2	2	2	2	2	2	2	**Gammaproteobacteria**
**SG8-30**	1	1	1	2	0	1	1	1	1	1	1	1	1	1	1	1	1	1	1	1	1	1	1	1	1	1	1	1	1	1	1	1	1	1	1	0	1	**Gammaproteobacteria**
**SG8-47**	1	1	2	1	0	1	1	1	1	1	1	1	1	1	1	1	1	1	1	1	1	2	1	1	1	1	1	2	1	1	1	1	1	1	1	0	1	**Gammaproteobacteria**
**SG8-15**	1	0	1	2	1	0	0	0	0	0	2	1	1	0	0	1	1	1	1	0	1	2	1	0	1	1	1	1	1	1	1	1	1	1	0	1	1	**Gammaproteobacteria**
**SG8-11**	0	0	0	2	1	0	0	0	0	0	1	1	1	0	1	1	0	1	1	0	1	0	1	1	1	1	0	0	1	1	1	1	1	1	0	0	1	**Gammaproteobacteria**
**SG8-45**	1	1	1	2	1	1	1	1	0	1	0	1	1	1	2	1	1	0	1	1	1	1	1	2	1	1	1	1	1	1	1	1	1	1	0	1	1	**Gammaproteobacteria**
**SM23-46**	1	0	1	3	1	0	0	0	0	0	2	1	1	0	0	1	1	1	1	0	1	1	1	0	1	1	1	1	1	1	1	1	0	1	0	1	1	**Gammaproteobacteria**
**SM1-46**	1	0	1	3	1	0	0	0	0	0	2	1	1	0	0	1	1	1	1	0	1	1	1	0	1	1	1	1	1	1	1	1	0	1	0	1	1	**Gammaproteobacteria**
**SG8-50**	0	0	0	2	2	1	1	1	1	1	0	1	1	1	0	1	2	0	1	1	1	0	1	0	1	1	2	0	1	2	1	1	0	1	0	2	1	**Gammaproteobacteria**
**SG8-23**	1	1	2	2	1	1	1	1	1	1	1	1	0	1	2	1	1	1	0	1	1	2	1	2	1	1	1	2	1	1	0	1	0	1	1	1	1	**Gemmatiomonas**
**SG8-38-2**	1	1	0	1	0	1	1	1	1	1	0	1	1	1	2	1	1	0	2	1	2	0	1	2	1	1	1	0	1	0	1	1	0	1	1	1	1	**Gemmatiomonas**
**SG8-28**	1	0	0	3	1	0	0	0	1	0	1	1	1	0	1	1	1	0	1	0	1	0	1	1	1	1	1	1	1	1	1	1	0	1	1	1	1	**Gemmatiomonas**
**SG8-17**	0	1	0	0	1	1	1	1	0	1	1	1	1	1	0	1	1	1	1	1	1	0	1	0	1	1	1	0	1	0	1	1	2	1	0	0	1	**Gemmatiomonas**
**SM23-52**	1	0	1	2	2	0	0	0	1	0	1	1	1	0	1	1	1	1	1	0	1	1	1	1	1	1	1	1	1	1	1	1	0	1	1	1	1	**Gemmatiomonas**
**DG-56**	0	1	0	1	0	1	1	1	0	1	0	1	1	1	1	1	1	0	1	1	1	0	1	1	1	1	1	0	1	2	1	1	0	1	0	2	1	**KD3-62**
**SG8-38-1**	2	1	2	2	1	1	1	1	1	1	1	1	1	1	1	1	0	1	1	1	1	2	1	1	1	1	0	2	1	1	1	1	0	1	1	1	1	**Myxococcales**
**SG8-38**	1	1	1	2	1	1	1	1	1	1	1	1	1	1	0	1	1	1	1	1	1	1	1	0	1	1	1	1	1	1	1	1	1	1	1	0	1	**Myxococcales**
**SG8-3**	1	1	0	4	1	1	1	1	2	1	1	2	1	1	1	1	0	0	1	1	1	0	2	1	2	1	1	0	1	1	1	1	2	2	2	1	2	**Nitrospira**
**SG8-35-4**	1	0	0	3	1	0	0	0	1	0	2	1	1	0	1	1	1	2	1	0	1	0	1	1	1	1	1	0	1	2	1	1	1	1	1	1	1	**Nitrospira/ like Thermodesulfovibrio**
**SG8-35-1**	1	0	0	3	1	1	1	1	1	1	2	1	1	1	0	1	1	2	1	1	0	0	1	0	1	1	1	0	1	1	1	3	1	1	1	2	1	**Nitrospira/ like Thermodesulfovibrio**
**SM23-35**	1	1	0	1	1	1	1	1	1	1	2	1	1	1	0	1	1	2	1	1	2	0	1	0	1	1	1	0	1	0	1	1	1	1	1	1	1	**Nitrospira/ like Thermodesulfovibrio**
**SG8-24**	1	1	1	0	1	1	1	1	1	1	1	1	1	1	1	1	1	1	1	1	1	1	1	1	1	1	1	1	1	1	1	1	0	1	1	2	1	**OD1**
**DG-74-1**	1	1	0	1	1	1	1	1	1	1	1	1	1	1	1	1	1	2	2	1	1	1	1	1	1	1	1	0	1	0	1	1	0	1	1	0	1	**OD1**
**DG-74-2**	1	2	0	3	2	1	1	1	2	1	1	1	2	1	3	1	1	1	2	1	2	1	2	3	1	1	2	1	2	0	2	2	0	1	2	1	2	**OD1**
**DG-74-3**	1	1	0	1	1	1	1	1	1	1	1	1	1	1	1	1	1	1	1	1	1	1	1	1	1	1	1	1	1	0	1	1	0	1	1	1	1	**OD1**
**DG-72**	0	1	0	3	1	1	1	1	1	1	1	1	1	1	1	1	1	1	1	1	1	3	1	1	1	1	1	1	1	0	1	1	0	1	1	1	1	**OD1**
**DG-75**	0	1	0	1	1	1	1	1	1	2	0	1	1	1	1	1	1	1	0	1	1	0	1	1	1	1	1	0	1	0	1	1	0	1	1	0	1	**OP11**
**SM23-25**	1	1	1	3	0	1	1	1	0	1	2	1	1	1	1	1	1	0	1	1	1	1	1	1	1	1	1	1	1	0	1	1	0	1	0	0	1	**Plantomycetes**
**SM23-32**	1	1	1	2	0	1	1	1	0	1	1	1	1	1	1	1	1	1	1	1	1	1	1	1	1	1	1	1	1	0	1	1	0	1	0	0	1	**Plantomycetes**
**SM23-65**	1	1	1	2	0	1	1	1	0	1	0	0	1	1	2	0	1	0	1	1	1	1	1	2	0	1	1	1	1	0	1	1	0	0	0	0	1	**Plantomycetes**
**DG-20**	0	2	1	2	0	2	2	2	1	2	2	1	1	2	2	1	1	0	1	2	1	1	1	2	1	1	1	1	1	0	1	1	0	1	1	0	1	**Plantomycetes**
**DG-23**	1	1	1	2	1	1	1	1	1	1	2	1	1	1	1	1	1	1	1	1	1	1	1	1	1	1	1	1	1	1	1	1	0	1	1	1	1	**Plantomycetes**
**DG-58**	1	1	1	1	0	1	1	1	1	1	0	1	1	1	1	1	1	0	1	1	1	1	1	1	1	1	1	1	1	0	1	1	0	1	1	0	1	**Plantomycetes**
**SG8-4**	0	1	2	0	1	1	1	1	1	1	0	1	1	1	1	1	1	2	1	1	1	1	1	1	1	1	1	1	1	0	1	1	1	1	1	0	1	**Plantomycetes/Phycisphaerae**
**SM23-30**	1	1	1	1	1	1	1	1	0	1	1	1	1	1	1	1	1	0	1	1	1	1	1	1	1	1	1	1	1	0	1	1	0	1	1	0	1	**Plantomycetes/Phycisphaerae**
**SM23-33**	1	1	1	0	1	1	1	1	1	1	0	1	1	1	1	1	1	1	1	1	1	1	1	1	1	1	1	1	1	1	1	1	0	1	1	0	1	**Plantomycetes/Phycisphaerae**
**SM23-32-1**	1	1	2	1	1	1	1	2	0	1	0	1	1	1	1	1	1	0	1	1	1	2	1	1	1	1	1	2	1	0	1	1	1	1	1	0	1	**Plantomycetes/Phycisphaerae**
**SM1-79**	1	1	0	1	1	1	1	1	0	1	1	1	1	0	1	1	1	0	1	1	1	0	1	1	1	1	1	0	1	0	1	1	1	1	0	0	1	**Plantomycetes/Phycisphaerae**
**DG-61**	1	1	1	1	1	1	1	1	0	1	1	0	1	1	1	0	1	1	1	1	1	1	0	1	0	1	1	1	0	2	1	1	0	0	0	1	0	**Spirochete**
**DG-26**	1	1	1	2	2	2	1	1	1	1	1	2	2	1	1	1	1	1	2	1	3	1	2	1	2	2	1	1	2	1	2	1	0	2	1	0	2	**TA06**
**DG-24**	1	1	1	2	0	1	1	1	1	1	0	1	1	1	0	1	1	0	1	1	1	1	1	0	1	1	1	1	1	1	1	1	0	1	1	0	1	**TA06**
**SM23-40**	1	1	1	2	1	1	1	1	1	1	1	1	1	1	1	1	1	1	1	1	1	1	1	1	1	1	1	1	1	1	1	1	1	1	1	1	1	**TA06**
**SM1-40**	1	1	1	2	1	1	1	1	1	1	1	1	1	1	1	1	1	1	1	1	1	1	1	1	1	1	1	1	1	1	1	1	1	1	1	1	1	**TA06**
**DG-78**	0	0	1	1	1	0	0	0	0	0	1	1	1	0	1	1	1	1	1	0	1	1	1	1	1	0	1	1	1	1	1	1	0	1	1	0	1	**TA06**
**DG-78-2**	1	1	1	1	1	1	1	1	1	1	0	1	1	1	1	1	1	0	1	1	1	1	1	1	1	1	1	1	1	1	1	1	0	1	1	1	1	**WOR-3**
**SM23-60**	1	0	1	1	1	1	1	1	1	1	2	1	1	1	2	1	1	2	1	1	1	1	1	2	1	1	1	1	1	1	1	2	0	1	1	1	1	**WOR-3**
**SM23-42**	1	1	1	1	1	1	1	1	1	1	1	1	1	1	1	1	1	1	1	1	1	1	1	1	1	1	1	1	1	1	1	1	0	1	1	0	1	**WOR-3**
**SM1-77**	0	0	0	1	0	1	0	0	0	0	1	1	0	1	0	1	1	0	0	0	0	0	1	0	1	1	1	0	0	0	0	1	0	1	0	0	1	**WOR-3**
**DG-54-3**	2	1	2	1	1	1	1	2	2	1	2	1	1	1	2	1	2	0	1	1	1	2	1	2	1	1	2	2	1	0	1	1	0	1	2	0	1	**WOR-1**
**DG-63**	1	1	1	2	2	1	1	1	1	1	1	1	1	1	1	1	1	1	1	1	1	1	1	1	1	1	1	1	1	1	1	1	1	1	1	2	1	**WS3**
**SM23-81**	1	1	1	2	1	1	1	1	1	1	2	1	1	1	1	1	1	1	1	1	1	1	1	0	1	1	1	1	1	1	1	1	0	1	1	1	1	**Zixibacteria**
**DG-27**	1	1	1	1	1	1	1	1	1	1	2	1	1	1	1	1	1	1	1	1	1	1	1	1	1	1	1	1	1	1	1	1	0	1	1	1	1	**Zixibacteria**
**SM23-73-3**	1	1	1	2	1	1	1	1	1	1	1	1	1	1	1	1	1	1	1	1	1	1	1	1	1	1	1	1	1	1	1	1	0	1	1	1	1	**Zixibacteria**
**SM23-73**	0	1	1	0	1	1	1	1	0	1	1	1	1	1	2	1	1	1	1	1	1	1	1	2	1	0	1	1	1	1	1	1	0	1	0	1	1	**Zixibacteria**
**SM23-73-2**	1	0	1	3	1	0	0	0	1	0	1	1	1	0	1	1	1	1	1	0	1	1	1	1	1	1	1	1	1	2	1	1	0	1	1	2	1	**Zixibacteria**
**sM23-57**	0	0	0	1	1	1	1	1	1	0	0	1	1	1	0	1	2	0	2	1	1	0	1	0	1	1	1	0	1	1	1	0	0	1	1	0	1	**KSB1**
**SM1-73**	1	1	1	1	1	1	1	1	0	1	1	1	1	1	1	1	1	1	1	1	1	1	1	1	1	1	1	1	1	1	1	1	0	1	0	0	1	**Zixibacteria**

Table S4. General characteristics of all bacterial genomic bins.

bin	# of scaffolds	# of genes	Total length	Taxonomy	longest scaffold	%GC
SG8-41	147	3889	3747630	Betaproteobacteria	202275	62.8839161
SG8-39	233	3307	3137175	Betaproteobacteria	98955	68.07786462
SG8-40	260	2744	2433805	Betaproteobacteria	33740	60.29198386
SG8-19	437	4886	4668865	Chloroflexi;Anaerolineae	119936	51.5720209
SG8-51-3	204	2080	1759043	Chloroflexi;Dehalococcoidia	51164	50.38485505
SG8-35-2	292	4049	3649796	Deltaproteobacteria	185839	46.07937708
SG8-35	206	2740	2591650	Deltaproteobacteria	58461	48.83851128
SG8-13	125	3814	3800553	Deltaproteobacteria	155941	56.91408943
SG8-31	266	6175	5968366	Gammaproteobacteria	339997	62.582524
SG8-11	464	4390	3882126	Deltaproteobacteria	12335	46.90937109
SG8-30	113	2780	2820123	Gammaproteobacteria	111313	68.74331424
SG8-15	302	3171	2741304	Gammaproteobacteria	36497	47.90531801
SG8-47	197	2931	2696743	Gammaproteobacteria	55750	60.61424087
SG8-45	98	2517	2283082	Deltaproteobacteria	97626	55.9487758
SG8-50	366	4182	3645285	Deltaproteobacteria	50482	57.24378482
SG8-28	430	3820	3856507	Gemmatiomonas	46899	67.17807476
SG8-17	456	3949	3691854	Gemmatiomonas	61136	59.95311963
SG8-23	310	3180	3209539	Gemmatiomonas	55555	68.08411909
SG8-38-2	307	3039	2814545	Gemmatiomonas	143644	61.53572883
SG8-38-1	234	3900	3848836	Myxococcales	133715	63.03172121
SG8-38	105	3453	3623660	Myxococcales	147758	63.40565039
SG8-3	733	6668	5957751	Nitrospira	34170	49.97484688
SG8-35-1	357	3808	3333117	Nitrospira/ like Thermodesulfovibrio	79085	45.81661615
SG8-35-4	292	3028	2692108	Nitrospira/ like Thermodesulfovibrio	33354	46.42563616
SG8-24	67	1231	1116563	OD1	62749	61.43724338
SG8-4	479	4333	4458874	Plantomycetes/Phycisphaerae	39007	54.80430706
**SMTZ**						
61	286	4504	4241461	Deltaproteobacteria	62586	56.08104922
62	403	5339	5784682	Bacteroides	142533	45.75321013
62-1	315	4729	5049351	Bacteroides	73049	40.02475855
62	307	2552	2576725	Bacteroides	142533	45.23576857
51	306	2831	2764529	BRC1	55748	59.78324458
31	300	3403	2821327	KSB1	34851	40.10903135

63	171	4302	4281581	Chloroflexi;Anaerolineae	197251	50.90573624
84	362	3995	3734778	Chloroflexi;Anaerolineae	50007	59.28764008
28-2	107	1566	1469117	Chloroflexi;Dehalococcoidia	53467	63.74960453
28-1	89	845	732023	Chloroflexi;Dehalococcoidia	22368	63.64467893
39	67	1253	1126604	Chlymidae	49818	26.25059689
46	263	3271	2826034	Gammaproteobacteria	54660	61.37935677
46	182	2960	2616358	Gammaproteobacteria	54660	61.64499321
52	263	4251	4296877	Gemmatiomonas	93174	65.49586914
35	140	1633	1416608	Nitrospira/ like Thermodesulfovibrio	46553	46.74738163
25	418	3775	3770408	Planctomyce	59260	66.54003754
32	237	2252	2326512	Planctomyce	27318	69.17579796
65	212	1773	1825793	Planctomyce	34095	62.18587834
30	296	3613	4021226	Plantomycetes/Phycisphaer ae	50768	50.74162926
33	322	3284	3426709	Plantomycetes/Phycisphaer ae	41894	67.77668015
32-1	317	2856	3094560	Plantomycetes/Phycisphaer ae	44002	68.27649076
79	241	2878	2952060	Plantomycetes/Phycisphaer ae	89332	50.32068985
40	189	2890	2883088	TA06	79299	60.2772819
40	197	2541	2559446	TA06	79299	60.30302857
60	264	3104	3369763	WOR-3	63010	47.40923541
42	59	2515	2532789	WOR-3	339863	46.21966529
77	150	1502	1269144	WOR-3	207686	45.77445293
72	137	2999	3297702	WOR-2	100134	44.99863358
29	173	2251	2222379	WOR-2	59882	41.69306428
81	99	2856	3074913	Zixibacteria	205519	52.81730816
57	285	2369	2350405	KSB1	81445	46.3441561
73-2	124	2356	2272909	Zixibacteria	70866	42.32916193
73	147	2480	2223615	Zixibacteria	46423	43.9719748
73-3	90	1939	1882756	Zixibacteria	85050	45.15644585
73	163	1657	1441479	Zixibacteria	46423	43.01709059

Deep

18	129	1101	936096	Chloroflexi;Dehalococcoidia	23208	53.88107428
22	147	1356	1135001	Chloroflexi;Dehalococcoidia	21435	63.45787596
28	199	1722	1418734	Deltaproteobacteria	13759	39.03181243
60	128	1665	1357456	Deltaproteobacteria	44074	39.04985039
40	88	2555	2338617	Gammaproteobaciteria/Coxi ella	273781	39.87171198
56	144	1261	1238975	KD3-62	32772	64.88600714
74-2	79	1584	1286363	OD1	198637	33.12639861
72	11	874	717349	OD1	207750	34.59752037
74-1	13	655	584570	OD1	146628	38.44891542

74-3	21	676	543780	OD1	114447	36.83598314
75	53	581	473645	OP11	32291	40.78200155
20	331	2799	2821569	Plantomycetes	32318	67.44076267
23	173	2069	2020164	Plantomycetes	41302	52.37501957
58	220	1720	1746620	Plantomycetes	32019	62.06938273
61	431	4757	4307950	Spirochete	56909	46.72409844
26	135	1727	1682905	TA06	48904	52.04594667
24	121	2291	2323720	TA06	131201	60.60071296
78-2	95	2768	2562206	WOR-3	105856	42.19918887
78	173	2196	2090507	TA06	50145	40.11276662
54-3	279	3638	3303329	WOR-1	114009	44.77026581
63	83	3190	3519707	WS3	259192	54.98622834
27	133	1142	1101969	Zixibacteria	25637	53.61606427

Table S5. Table showing the abundance and identification of genes involved in protein degradation in the genomic bins in this study. The pfam and EC numbers used to search the annotations are provided. The number in each cell represents the number of that particular gene family that was identified.

Table S6. A complete list of the abundance and specific genes in the genomic bins involved in degradation of organic carbon compounds identified by comparison to the CAZy database. The number represents the number of that particular gene that was found in that bin.

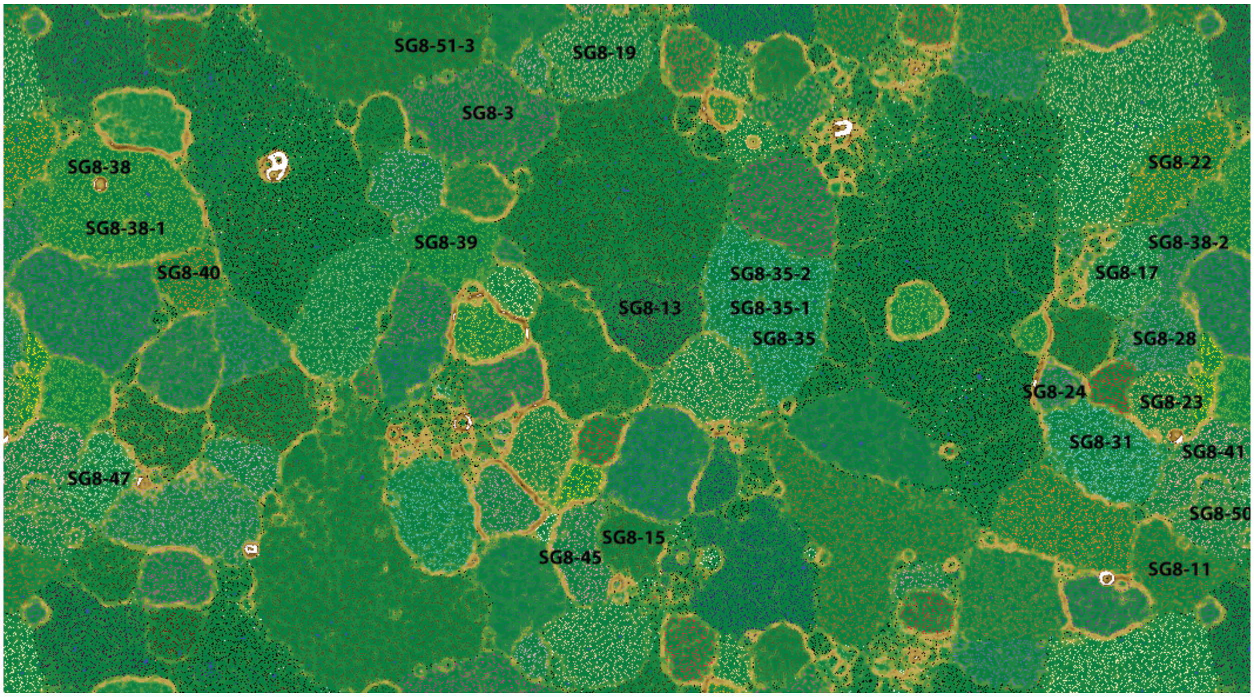

Figure S1. Tetra-nucleotide ESOM binning map of shallow assembly. Each data points is a 5kb portion of DNA sequence. Seperation of data points are visible as brown lines (background color) which delineates clustering of sequences by the mapping. The manually delineated bins are colored and labeled. Noticed that in a few cases (SG8-35, 35-1, 35-2, and SG8-38 and 38-1) multiple closely-related bins falls within one large cluster on this map. These bins were found to contain more than one genome and were therefore, further delineated based on differential coverage plots.

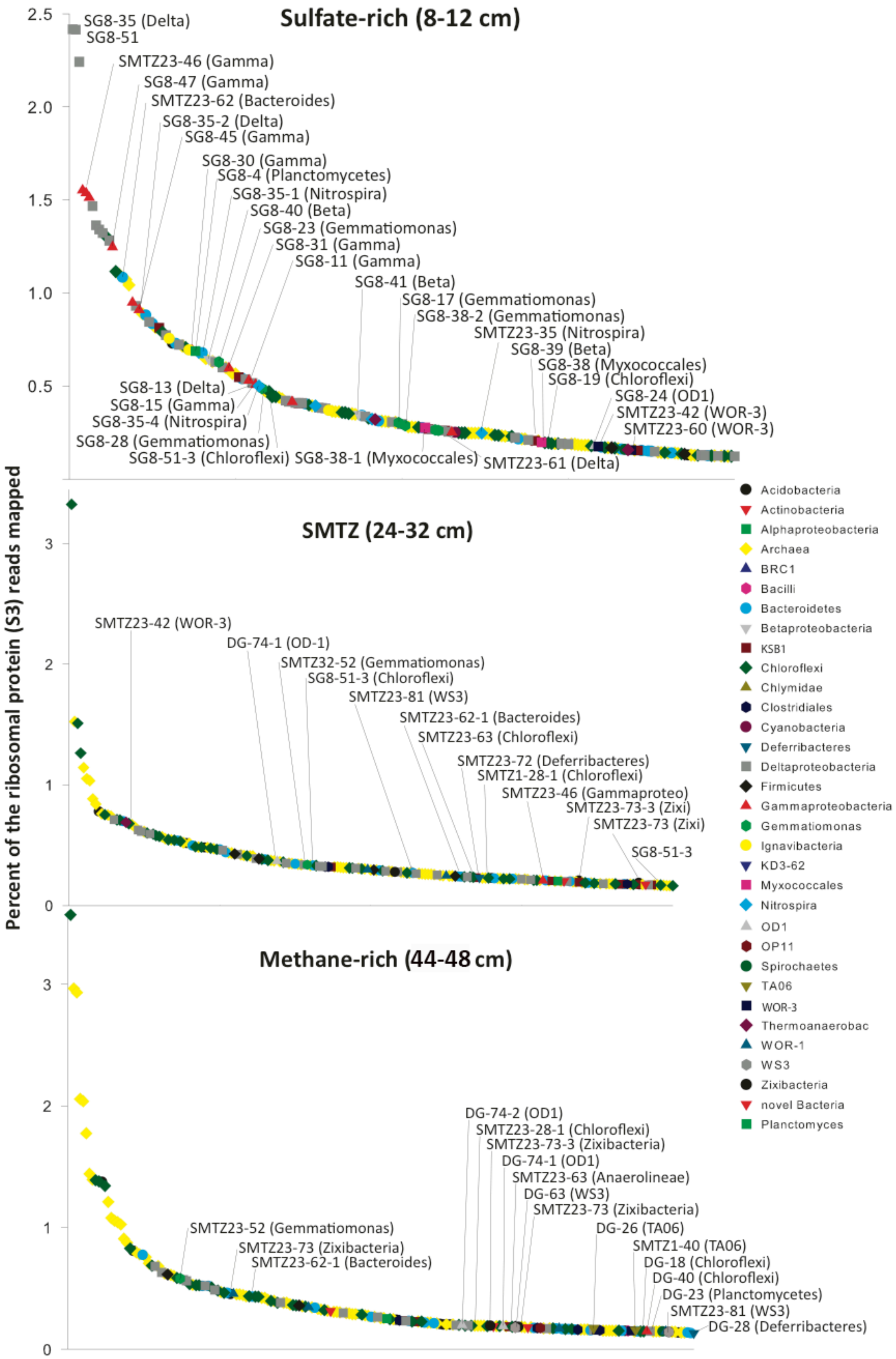
Sulfate-rich (8-12 cm)
SMTZ (24-32 cm)
Methane-rich (44-48 cm)
Percent of the ribosomal protein (S3) reads mapped
2.5
2.0
1.5
1.0
0.5
3
2
1
0
SG8-35 (Delta)
SG8-51
SMTZ23-46 (Gamma)
SG8-47 (Gamma)
SMTZ23-62 (Bacteroides)
SG8-35-2 (Delta)
SG8-45 (Gamma)
SG8-30 (Gamma)
SG8-4 (Planctomycetes)
SG8-35-1 (Nitrospira)
SG8-40 (Beta)
SG8-23 (Gemmatiomonas)
SG8-31 (Gamma)
SG8-11 (Gamma)
SG8-41 (Beta)
SG8-17 (Gemmatiomonas)
SG8-38-2 (Gemmatiomonas)
SMTZ23-35 (Nitrospira)
SG8-39 (Beta)
SG8-38 (Myxococcales)
SG8-19 (Chloroflexi)
SG8-24 (OD1)
SMTZ23-42 (WOR-3)
SMTZ23-60 (WOR-3)
SG8-13 (Delta)
SG8-15 (Gamma)
SG8-35-4 (Nitrospira)
SG8-28 (Gemmatiomonas)
SG8-51-3 (Chloroflexi)
SG8-38-1 (Myxococcales)
SMTZ23-61 (Delta)
SMTZ23-42 (WOR-3)
DG-74-1 (OD-1)
SMTZ32-52 (Gemmatiomonas)
SG8-51-3 (Chloroflexi)
SMTZ23-81 (WS3)
SMTZ23-62-1 (Bacteroides)
SMTZ23-63 (Chloroflexi)
SMTZ23-72 (Deferribacteres)
SMTZ1-28-1 (Chloroflexi)
SMTZ23-46 (Gammaproteo)
SMTZ23-73-3 (Zixi)
SMTZ23-73 (Zixi)
SG8-51-3
DG-74-2 (OD1)
SMTZ23-28-1 (Chloroflexi)
SMTZ23-73-3 (Zixibacteria)
DG-74-1 (OD1)
SMTZ23-63 (Anaerolineae)
DG-63 (WS3)
SMTZ23-73 (Zixibacteria)
DG-26 (TA06)
SMTZ1-40 (TA06)
DG-18 (Chloroflexi)
DG-40 (Chloroflexi)
DG-23 (Planctomycetes)
SMTZ23-81 (WS3)
DG-28 (Deferribacteres)
SMTZ23-52 (Gemmatiomonas)
SMTZ23-73 (Zixibacteria)
SMTZ23-62-1 (Bacteroides)
Acidobacteria
Actinobacteria
Alphaproteobacteria
Archaea
BRC1
Bacilli
Bacteroidetes
Betaproteobacteria
KSB1
Chloroflexi
Chlymidae
Clostridiales
Cyanobacteria
Deferribacteres
Deltaproteobacteria
Firmicutes
Gammaproteobacteria
Gemmatiomonas
Ignavibacteria
KD3-62
Myxococcales
Nitrospira
OD1
OP11
Spirochaetes
TA06
WOR-3
Thermoanaerobac
WOR-1
WS3
Zixibacteria
novel Bacteria
Planctomyces

Figure S2. Fig. Abundances of top genotypes in the SMTZ (24-32 cm) and methane-rich (44-48 cm) layers of the sediment profiles based on the number of reads that map to all the genes for ribosomal protein S3. Those that are represented in the genomic bins are labeled.

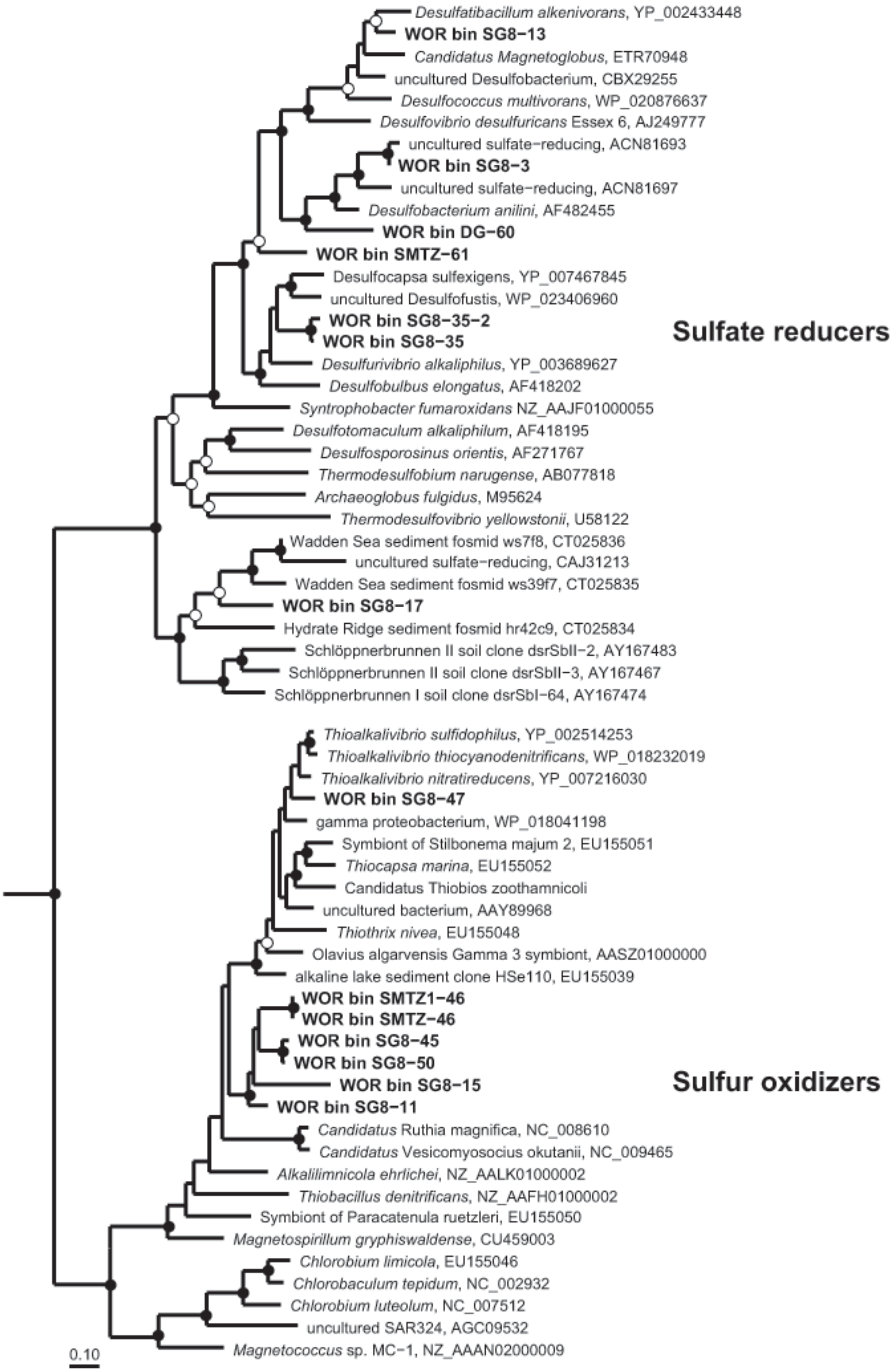
Desulfatibacillum alkenivorans, YP_002433448
WOR bin SG8−13
Candidatus Magnetoglobus, ETR70948
uncultured Desulfobacterium, CBX29255
Desulfococcus multivorans, WP_020876637
Desulfovibrio desulfuricans Essex 6, AJ249777
uncultured sulfate−reducing, ACN81693
WOR bin SG8−3
uncultured sulfate−reducing, ACN81697
Desulfobacterium anilini, AF482455
WOR bin DG−60
WOR bin SMTZ−61
Desulfocapsa sulfexigens, YP_007467845
uncultured Desulfofustis, WP_023406960
WOR bin SG8−35−2
WOR bin SG8−35
Sulfate reducers
Desulfurivibrio alkaliphilus, YP_003689627
Desulfobulbus elongatus, AF418202
Syntrophobacter fumaroxidans NZ_AAJF01000055
Desulfotomaculum alkaliphilum, AF418195
Desulfosporosinus orientis, AF271767
Thermodesulfobium narugense, AB077818
Archaeoglobus fulgidus, M95624
Thermodesulfovibrio yellowstonii, U58122
Wadden Sea sediment fosmid ws7f8, CT025836
uncultured sulfate−reducing, CAJ31213
Wadden Sea sediment fosmid ws39f7, CT025835
WOR bin SG8−17
Hydrate Ridge sediment fosmid hr42c9, CT025834
Schlöppnerbrunnen II soil clone dsrSbII−2, AY167483
Schlöppnerbrunnen II soil clone dsrSbII−3, AY167467
Schlöppnerbrunnen I soil clone dsrSbI−64, AY167474
Thioalkalivibrio sulfidophilus, YP_002514253
Thioalkalivibrio thiocyanodenitrificans, WP_018232019
Thioalkalivibrio nitratireducens, YP_007216030
WOR bin SG8−47
gamma proteobacterium, WP_018041198
Symbiont of Stilbonema majum 2, EU155051
Thiocapsa marina, EU155052
Candidatus Thiobios zoothamnicoli
uncultured bacterium, AAY89968
Thiothrix nivea, EU155048
Olavius algarvensis Gamma 3 symbiont, AASZ01000000
alkaline lake sediment clone HSe110, EU155039
WOR bin SMTZ1−46
WOR bin SMTZ−46
WOR bin SG8−45
WOR bin SG8−50
WOR bin SG8−15
Sulfur oxidizers
WOR bin SG8−11
Candidatus Ruthia magnifica, NC_008610
Candidatus Vesicomyosocius okutanii, NC_009465
Alkalilimnicola ehrlichei, NZ_AALK01000002
Thiobacillus denitrificans, NZ_AAFH01000002
Symbiont of Paracatenula ruetzleri, EU155050
Magnetospirillum gryphiswaldense, CU459003
Chlorobium limicola, EU155046
Chlorobaculum tepidum, NC_002932
Chlorobium luteolum, NC_007512
uncultured SAR324, AGC09532
Magnetococcus sp. MC−1, NZ_AAAN02000009
0.10

Figure S3. Phylogenetic tree of concatenated *dsrAB* genes within bacteria genomic bins from this study. Closed and open circles represent maximum likelihood (ProML, ARB package) bootstrap value >75 and >50, respectively.

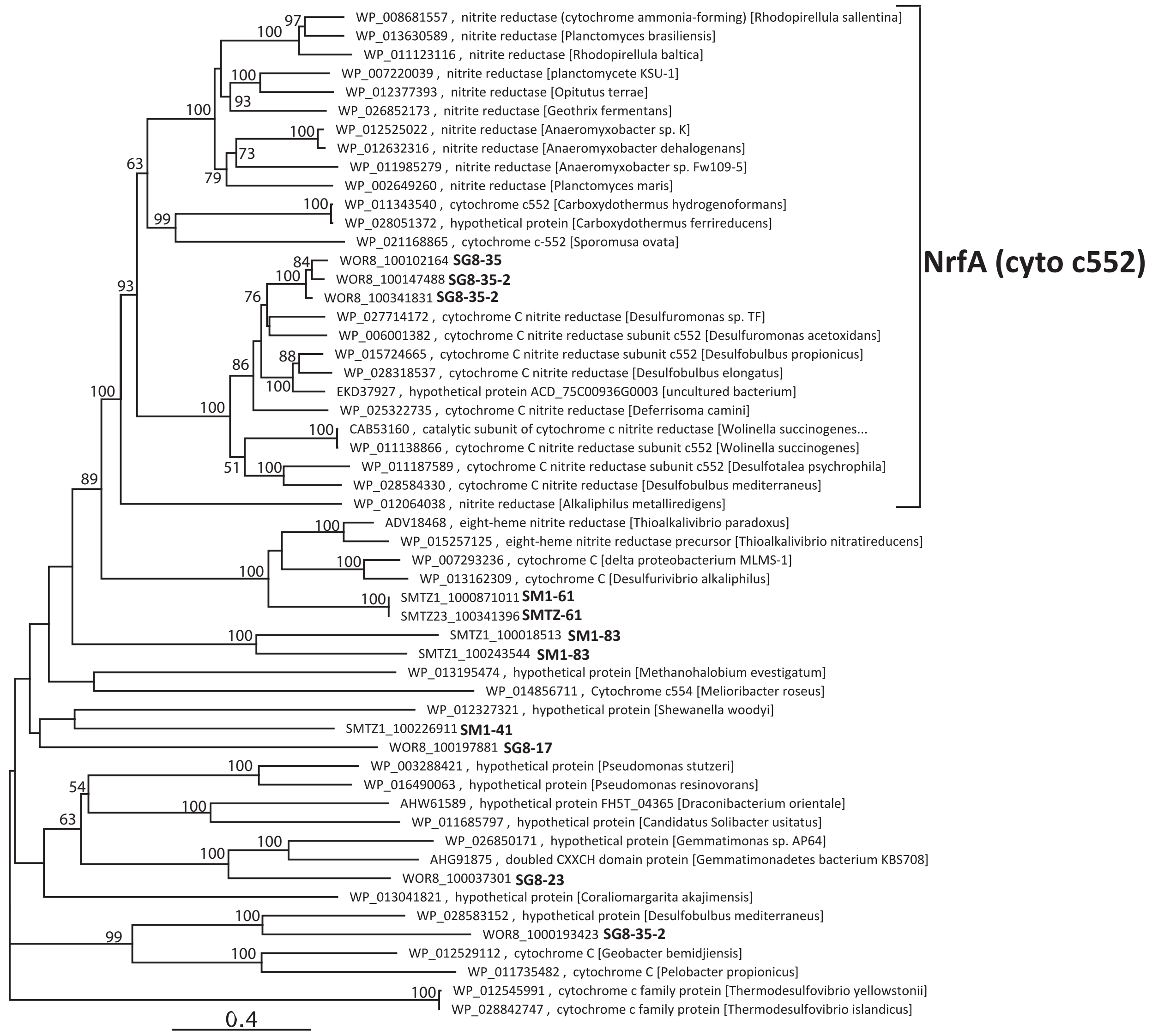

Figure S4. Phylogenetic tree of WOR proteins annotated at formate-dependent nitrite-reductase (periplasmic cyctochrome c552), NrfA. Cluster of sequences considered to be involved in DNRA are delineated on the right (Welsh et al. 2014). Genomic bin designations are shown in bold.
